# Bacterial Extracellular Vesicles (BEVs) Derived from *Chryseobacterium* Inhibit Dengue Virus Infection by Disrupting Its Structural Integrity

**DOI:** 10.1002/jev2.70302

**Published:** 2026-06-23

**Authors:** Yaqi Gao, Lijian Zhang, Tianci Zhang, Qiufeng Yao, Ruifang Gao, Yue Wang, Yunpeng Zhao, Tingting Zhou, Jikuai Chen, Xing Zhang, Hao Ren, Yongzhe Zhu, Ping Zhao, Zhongtian Qi, Li Luo, Zhaoling Qin

**Affiliations:** ^1^ Department of Microbiology, Shanghai Key Laboratory of Medical Biodefense, Faculty of Naval Medicine Naval Medical University Shanghai China; ^2^ Shanghai Key Laboratory of Bio‐energy Crops, Center of Plant Science, School of Life Sciences Shanghai University Shanghai China; ^3^ Department of Stem Cell and Regeneration Medicine, Translational Medicine Research Center Naval Medical University Shanghai China; ^4^ Department of Pharmaceutical Analysis, School of Pharmacy Naval Medical University Shanghai China; ^5^ Department of Health Toxicology, Faculty of Naval Medicine Naval Medical University Shanghai China

**Keywords:** antiviral mechanism, bacterial extracellular vesicles, *Chryseobacterium*, dengue virus, enveloped virus

## Abstract

Dengue virus (DENV) infection poses a significant global health threat, and current prevention and treatment strategies are limited by challenges of lacking effective mosquito control measures and antibody‐dependent enhancement. This study reports that bacterial extracellular vesicles (BEVs) secreted by a soil bacterium *Chryseobacterium aquifrigidense* M24 exhibit potent anti‐DENV activity by triggering the structural disintegration of DENV particles prior to cellular entry in a dose‐dependent manner. Mechanistic investigations revealed that BEVs interact with the viral envelope, inducing premature membrane fusion. This process is characterized by reduced membrane fluidity and irreversible lipid rearrangement, leading to a significant increase in particle density, as shown by iodixanol gradient ultracentrifugation. The proposed ‘fusion‐triggered structural disruption’ is further supported by the induction of aberrant E protein oligomerization and morphological changes observed via transmission electron microscopy. This mechanism is specific to enveloped viruses, as BEVs showed no effect on non‐enveloped Enterovirus 71. Crucially, this BEV‐mediated inactivation extends to other enveloped viruses, including HCV, WNV and YFV, indicating broad‐spectrum potential. Our findings reveal a previously unexplored function of BEVs as virucidal agents, proposing a new ‘virus‐destructor’ strategy that contrasts with conventional fusion inhibitors and offering promising avenues for developing broad‐spectrum antiviral drugs.

## Graphical abstract

This study elucidates a novel antiviral mechanism of bacterial BEVs, which are shown to inactivate viruses by triggering membrane fusion, subsequent lysis and causing structural collapse. This direct virucidal action presents a promising broad‐spectrum strategy against diverse enveloped viruses.

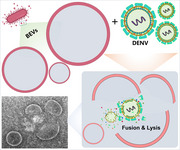

## 1 Introduction

Dengue virus (DENV) is a globally distributed arbovirus belonging to the genus *Flavivirus* within the family *Flaviviridae* (Hotta 1952; Kalimuddin et al. 2025; Paz‐Bailey et al. 2024). It comprises four serotypes (DENV‐1 to DENV‐4). DENV infection can lead to a spectrum of clinical manifestations, ranging from mild dengue fever to severe diseases such as dengue hemorrhagic fever and dengue shock syndrome (Rigau‐Perez et al. 1998). Recent studies have also indicated the neuroinvasive potential of DENV infection (Trivedi and Chakravarty 2022). Its infection is primarily transmitted through the bites of *Aedes aegypti *and *Aedes albopictus* mosquitoes and is endemic in tropical and subtropical regions (Kristan et al. 2025). According to World Health Organization (WHO) statistics, approximately 3.8 billion people in about 129 countries are at risk of DENV infection, with an estimated 100 to 400 million cases occurring annually. The global number of reported infections reached 14 million in 2024 (Haider et al. 2025). The mortality rate among hospitalized patients who develop severe disease is approximately 1%.

Currently, two tetravalent live‐attenuated vaccines (Dengvaxia and Qdenga) have been approved for marketing, and another candidate vaccine, TV003/TV005, is in Phase III clinical trials (Anumanthan et al. 2025; Kallas et al. 2024). However, due to the limited cross‐protection between different serotypes and the phenomenon of antibody‐dependent enhancement (ADE) (Rothman and Ennis 1999), which increases the risk of more severe disease upon secondary infection with a heterologous serotype, and coupled with the lack of effective specific antiviral drugs, dengue virus infection remains a major global public health challenge.

DENV is an enveloped virus containing a single positive‐stranded RNA genome of approximately 11 kb. The genome consists of 5' and 3' untranslated regions (UTRs) and a single open reading frame (ORF). The ORF encodes a polyprotein that is cleaved by host and viral proteases into three structural proteins, the capsid protein (C), membrane protein (prM/M) and envelope protein (E) and seven non‐structural proteins (NS1, NS2A, NS2B, NS3, NS4A, NS4B and NS5) (Malavige and Ogg 2024). The virus particle is spherical, approximately 45–55 nm in diameter, with an outer host‐derived lipid envelope embedded with the E and prM/M proteins (Perera and Kuhn 2008). The E protein is the key antigen for virus entry, responsible for binding to specific receptors on host cells and mediating entry via clathrin‐dependent or ‐independent endocytosis (I. M. Yu et al. 2008). Following endocytosis, the acidic environment of the endosome triggers conformational changes in the E protein, mediating fusion between the viral envelope and the endosomal membrane (Modis et al. 2004), thereby releasing the viral RNA into the cytoplasm.

Outer membrane vesicles (OMVs) are nano‐scale vesicular structures secreted mainly by Gram‐negative bacteria, originating from the budding of the outer membrane (Schwechheimer and Kuehn 2015; Toyofuku et al. 2019). Structurally and functionally similar to eukaryotic exosomes, OMVs play important roles in bacterial communication, biofilm formation and pathogenicity, and are regarded as natural and functional nanomaterials (Sartorio et al. 2021). Recent studies increasingly suggest that OMVs hold significant application potential not only in antibacterial therapy, as active antimicrobial agents or delivery vehicles for antibiotics, but also in the development of vaccines or immune adjuvants (Q. Q. Feng et al. 2022; Huang et al. 2022; Micoli et al. 2024).

It has been reported that *Bacillus subtilis* LjM2, isolated from aged dry citrus peel (Chenpi), secretes a specific protease, CPAVM1, which directly cleaves viral proteins, thereby enabling its culture supernatant to potently inhibit influenza A virus (IAV) infection(J. Li et al. 2024). Additionally, *Chromobacterium* species isolated from the midgut of *Aedes aegypti* produce antiviral effectors (CbAE‐1 and CbAE‐2), which disrupt the viral envelope structure through their lipase activity, resulting in broad‐spectrum anti‐arbovirus activity(X. Yu et al. 2022). Given that environmental microbes from sources such as soil and water are even more diverse and represent a rich source of natural products, we established a screening pipeline for antiviral activity against DENV based on similar approaches. In our preliminary screen for anti‐DENV drug candidates, we discovered that the culture supernatant of a soil‐derived *Chryseobacterium* *aquifrigidense* (*C. aquifrigidense*) strain M24, exhibited potent antiviral activity *in vitro*. Further investigation identified bacterial extracellular vesicles (BEVs) secreted by the bacterium as the primary active component responsible for inhibiting DENV infection. In‐depth mechanistic studies revealed that these BEVs interact directly with DENV virions, disrupting viral integrity. This study systematically elucidates the antiviral mechanism of BEVs against DENV and discusses their potential applications and challenges. It offers a new perspective and scientific basis for addressing mosquito‐borne viral diseases and developing antiviral agents that target viral structure disruption.

## 2 Materials and Methods

### 2.1 Bacterial Culture

The bacterium (*C. aquifrigidense* M24) used in this study was isolated from a soil sample. The bacterium was cultured in Yeast Extract‐Bacterial tryptone (YEB) liquid medium [yeast extract, 1.0 g; bacterial tryptone, 5.0 g; MgSO_4_·H_2_O, 0.5 g; sucrose, 5.0 g; distilled water, 1 liter] at 28°C with shaking at 200 rpm for overnight and then harvested. To obtain bacterial supernatant, the bacterial cultures were collected at stationary phase (approximately 16–18 h), centrifuged at 10,000 × g for 40 min at 4°C, and then filtered through a 0.22 µm filters membrane (Merck, SLGP033R) to remove bacterial cells.

### 2.2 Bacterial Extracellular Vesicles (BEVs) Preparation and Purification

Crude BEVs were extracted from the filtered supernatant via ultracentrifugation. Briefly, the bacterial supernatant was ultracentrifuged at 150,000 × g for 3 h at 4°C using a swing‐bucket rotor (P40ST, Hitachi, Japan). The pellet was resuspended in sterile PBS to obtain crude BEVs. For further purification, the crude preparation was subjected to 10%‐60% (w/v) iodixanol (Sigma, D1556‐250ML) density gradient ultracentrifugation in a P40ST rotor (150,000 × g, 4°C, 3 h). After centrifugation, ten 1.0‐ml fractions were collected sequentially from the top. Fractions enriched with BEVs were subjected to another round of ultracentrifugation. The final pellet was resuspended in PBS and stored at 4°C. The particle size and number of the BEVs were measured by nanoparticle tracking analysis (NTA) (Malvern, UK).

### 2.3 FM4‐64 Staining of Bacteria and Confocal Microscopy

To visualize the morphological and divisional characteristics of bacterial cells clearly, the bacteria in the logarithmic growth phase were collected by centrifugation, resuspended in PBS and the concentration of the bacterial suspension was adjusted. A final concentration of 5 µM of the lipophilic dye FM4‐64 (MCE, HY‐103466) was added, followed by incubation at room temperature protected from light for 10 min. Unbound dye was removed, and the samples were incubated with 4’, 6’‐ diamidino‐2‐phenylindole (DAPI, Roche, Switzerland) to stain the nucleoids. Then, the samples were washed with PBS for three times. Finally, 10 µL of the stained bacterial suspension was placed on a glass slide and gently covered with a coverslip, taking care to avoid air bubbles. The samples were observed using a Zeiss LSM 900 confocal fluorescence microscope with a high‐magnification oil immersion objective, and the images were processed and photographed using the dedicated professional software.

### 2.4 Cells and Viruses

Human hepatoma Huh7 cells (SCSP‐526, Chinese Academy of Sciences, Shanghai, China) and African green monkey Vero cells (ATCC CCL‐81) were both cultured in complete Dulbecco's Modified Eagle's Medium (DMEM) containing 10% (v/v) heat‐inactivated fetal bovine serum (FBS) (Gibco BRL, USA) supplemented with 100 nmol/L non‐essential amino acids (NEAA), 1 mmol/L L‐glutamine, 100 µg/mL streptomycin and 100 U/mL penicillin at 37°C in a 5% CO_2_ incubator. *Aedes albopictus* mosquito (C6/36) cells (ATCC CRL‐1660) were cultured in RPMI‐1640 medium (Gibco, C11875500BT) supplemented with 10% FBS and antibiotics at 28°C in a 5% CO_2_ incubator.

Dengue virus serotype 2 (DENV‐2, strain Tr1751, kindly provided by Prof. Jing An from Capital Medical University, Beijing, China) was propagated in C6/36 mosquito cells at 28°C. Viral supernatants were clarified, concentrated, aliquoted and stored at −80°C until use. Viral titers were determined by plaque assay on Vero cells and are shown as plaque‐forming units (PFU) /mL (Liu et al. 2021). Other viruses used in the study were listed in supplementary supporting information.

### 2.5 Viral Infection and Immunofluorescence Assay

Huh7 cells were seeded in 96‐well plates (costar, 3590) one day before infection and grown to 70–80% confluence. Bacterial culture supernatants or BEVs were diluted with complete DMEM to the indicated concentrations (0.075%, 0.75%, 0.938%, 1.875%, 3.75%, 7.5% and 15% for culture supernatants, v/v) and mixed with DENV at a multiplicity of infection (MOI) of 1. As a control, YEB medium or PBS was diluted to match the highest concentration (15%, v/v for supernatants) and similarly mixed with DENV at an MOI of 1. The mixtures were then inoculated onto the cells and incubated for 1 h at 37°C. Subsequently, the cells were washed with PBS and then cultured in fresh complete medium. After 24 h, cells were subjected to immunofluorescence assay as previously described (Gao et al. 2015; Khakpoor et al. 2009). Briefly, cells were fixed with 4% paraformaldehyde for 15 min at RT and then permeabilized with 0.1% Triton X‐100 for 5 min at RT. After being blocked with 3% BSA (in PBS) for 2 h, the samples were stained with rabbit polyclonal antibody against the DENV C protein (GeneTex, GTX124247), followed by an Alex Fluor 488‐conjugated secondary antibody. Fluorescence microscopy was used for observation and calculation of the infection rate.

### 2.6 Viral RNA Extraction and Quantitative Reverse Transcription‐PCR (qRT‐PCR)

To assess the effect of BEVs on key steps during DENV life cycle, specific viral attachment, entry and replication assays were designed. Total cellular RNA was extracted using a commercial RNAiso Plus TRIzol (TaKaRa, 9109) according to the manufacturer's manual. The RNA concentration was determined using a BioTek spectrophotometer. One microgram of total RNA was used for cDNA synthesis using PrimeScript^TM^ RT Master Mix (TaKaRa, RRO36A). TB Green^TM^ Premix Ex Taq^TM^ II (TaKaRa) was used as the fluorescent dye. qRT‐PCR analysis was performed using specific primers targeting the DENV genome, with the GAPDH gene serving as an internal control, and the relative fold‐change of viral RNA level was calculated and analyzed relative to controls by using the 2^−△△Ct^ method as described previously (Qin et al. 2013). All the assays were performed using an ABI 7300 system (Applied Biosystems, Massachusetts, USA).

### 2.7 Western Blot Analysis

Western blot analysis was performed as previously described, but with some modifications (Qin et al. 2007; Qin et al. 2004). Briefly, cells or fractions were lysed on ice by using RIPA lysis buffer (Beyotime, P0013B) containing a protease inhibitor cocktail and 1% phenylmethylsulfonyl fluoride (PMSF). Total protein concentrations of the supernatants were determined using the BeyoBCA Protein Assay Kit (Beyotime, P0399S) according to the manufacturer's manual. Subsequently, the protein samples were separated using 12.5% (w/v) SDS‐polyacrylamide gel electrophoresis (PAGE) and transferred onto PVDF membranes (Millipore, USA) by using a Trans‐Blot apparatus (Bio‐Rad, USA). The proteins of interest were identified using rabbit anti‐DENV Capsid and NS3 polyclonal antibodies (GeneTex, GTX124252) and rabbit anti‐OmpA polyclonal antibody (Cusabio, CSB‐PA359226ZA01EOD), and detected visually using horseradish peroxidase (HRP)‐conjugated species‐specific secondary antibodies (Invitrogen, 31460). Immunoreactivity was visualized by enhanced chemiluminescence technique using SuperSignal West Pico chemiluminescent substrate (Thermo Scientific, 1863096 and 1863097).

### 2.8 Analysis of BEV‐DENV Interaction by Density Gradient Centrifugation

To elucidate the interaction between BEVs and DENV, an *in vitro* co‐incubation assay was performed, followed by density gradient ultracentrifugation. Briefly, for the co‐incubation group, 500 µL of purified BEVs (7.8×10^10^ particles) was incubated with 500 µL of crude DENV supernatant (∼1.5 × 10^6^ PFU) at room temperature for 1 h. For the virus‐only control, BEVs were replaced with an equal volume of PBS; for the BEV‐only control, DENV was replaced with an equal volume of PBS. After incubation, 1 mL of each sample (the BEV–DENV mixture, virus‐only control and BEV‐only control) was layered onto a 10%‐60% iodixanol gradient and subjected to ultracentrifugation. Following centrifugation, ten fractions were sequentially collected from the top of each gradient. Each fraction was analyzed for protein content using the BCA assay, and their densities in all three groups; viral titer, viral RNA level and the distribution of viral structural proteins (C and E) were determined in the BEV‐DENV mixture and DENV‐only control groups; and the distribution of the BEV marker protein OmpA was determined in the BEV‐DENV mixture and BEV‐only control groups.

### 2.9 Transmission Electron Microscopy (TEM) Observation

Different samples including DENV, BEVs, or their mixtures and mid‐log phase bacterial culture, were appropriately diluted and ten microliters of indicated solution was applied directly onto copper grids for 5 min, followed by wiped dry at room temperature. The grids were then negatively stained in the dark with 2% uranyl acetate or 2% phosphotungstic acid for 2 min. After being rinsed with PBS and air‐drying, samples were observed and analyzed between 80 and 120 kV using JEM‐1400 transmission electron microscope equipped with a JEOL sCMOS camera for morphological structure.

### 2.10 Viral RNA Exposure Assay

Different concentrations of BEVs were mixed with DENV (1.4 × 10^4^ PFU) in a total volume of 200 µL, followed by incubation at 37°C for 2 h. An equal volume of PBS served as the negative control. Then, 2 µL of RNase A (Transgen, GE101‐01) was added to the mixture and incubated at 37°C for an additional 1 h. Viral RNA was extracted using TRIzol, and RNA degradation was evaluated by qRT‐PCR as previously described (Y. Feng et al. 2023; X. Yu et al. Yu et al. 2022).

### 2.11 Triglyceride (TG) Quantification

TG content in fractions from the density gradient centrifugation was measured using a Triglyceride Assay Kit (Sigma, MAK040) as previously described (Qin et al. 2022). Briefly, the indicated fractions were incubated at 90°C for 30 min, and then its TG level was measured via an enzymatic method using a Synergy H1 multi‐mode microplate reader (BioTek, USA). The amount of TGs in the samples was calculated according to the standard curve obtained from the TG standards provided by the manufacturer.

### 2.12 Relative Quantification of Total Membrane Lipids

To quantitatively analyze the total membrane lipid content in each ultracentrifugation fraction, we employed the lipophilic fluorescent probe FM4‐64 for relative quantification (Taraska and Almers 2004). Briefly, 50 µL of each fraction sample was diluted with PBS to a final volume of 100 µL, followed by the addition of the FM4‐64 probe to a final concentration of 5 µM. The mixture was incubated at room temperature in the dark for 30 min to ensure sufficient incorporation of the probe into the membrane lipids. A blank control containing only PBS and an equal amount of the probe was prepared simultaneously. Fluorescence was measured using a Synergy H1 multi‐mode microplate reader. Given the spectral characteristics of FM4‐64, the excitation wavelength was set at 515 nm, and the emission fluorescence intensity was collected at 640 nm. For data analysis, the measured value from each sample was subtracted by the blank control value to eliminate background interference. The resulting relative fluorescence units (RFU) for each fraction represents the relative content of total membrane lipids.

### 2.13 Membrane Fusion Kinetics Assay

BEVs (6.52×10^11^ particles) were labeled with the lipophilic fluorescent probe octadecyl rhodamine B chloride (R18, MKbio, MX4013‐10MG) at a final concentration of 80 µg/mL. The labeling was performed on a shaker at room temperature for 1 h, followed by purification via ultracentrifugation (150,000 × g, 4°C, 3 h) as described above (Ohki et al. 1998). Subsequently, different concentrations of the R18‐labeled BEVs (64, 48, 16×10^6^ particles/µL) were mixed with DENV (1.4×10^4^ PFU). The fluorescence intensity was monitored in real‐time for a total duration of 180 min, with measurements taken every 4 min. The change in R18 fluorescence intensity were quantified to assess the occurrence rate and extent of membrane fusion between BEVs and DENV.

### 2.14 Membrane Fluidity Assay

Membrane fluidity was assessed using Prodan probe as previously described (Luna et al. 2023). Briefly, the DENV stock were diluted with PBS to 2.0 × 10^5^ PFU/mL, followed by the addition of 15 µM Prodan and incubation for 15 min at room temperature. Fluorescence intensity was measured for the labeled DENV treated with BEVs (16, 32, 48, 64×10^6^ particles/µL) or PBS using a Synergy H1 multi‐mode microplate reader, with the excitation wavelength set to 350 nm and emission intensities recorded at 440 and 490 nm. Quantitative analysis of membrane fluidity was performed using the Prodan generalized polarization (GP) index. The calculation formula is: Prodan GP = (I440 – I490)/ (I440 + I490).

### 2.15 Statistical Analysis

Data are presented as mean ± standard deviation (SD) from three independent experiments. Statistical analysis was performed using GraphPad Prism software. Differences between groups were assessed using Student's t‐test or one‐ or two‐way analysis of variance (ANOVA).

## 3 Results

### 3.1 Bacterial Supernatant of C. Aquifrigidense M24 Exhibits Potent Antiviral Activity In Vitro

**FIGURE 1:**
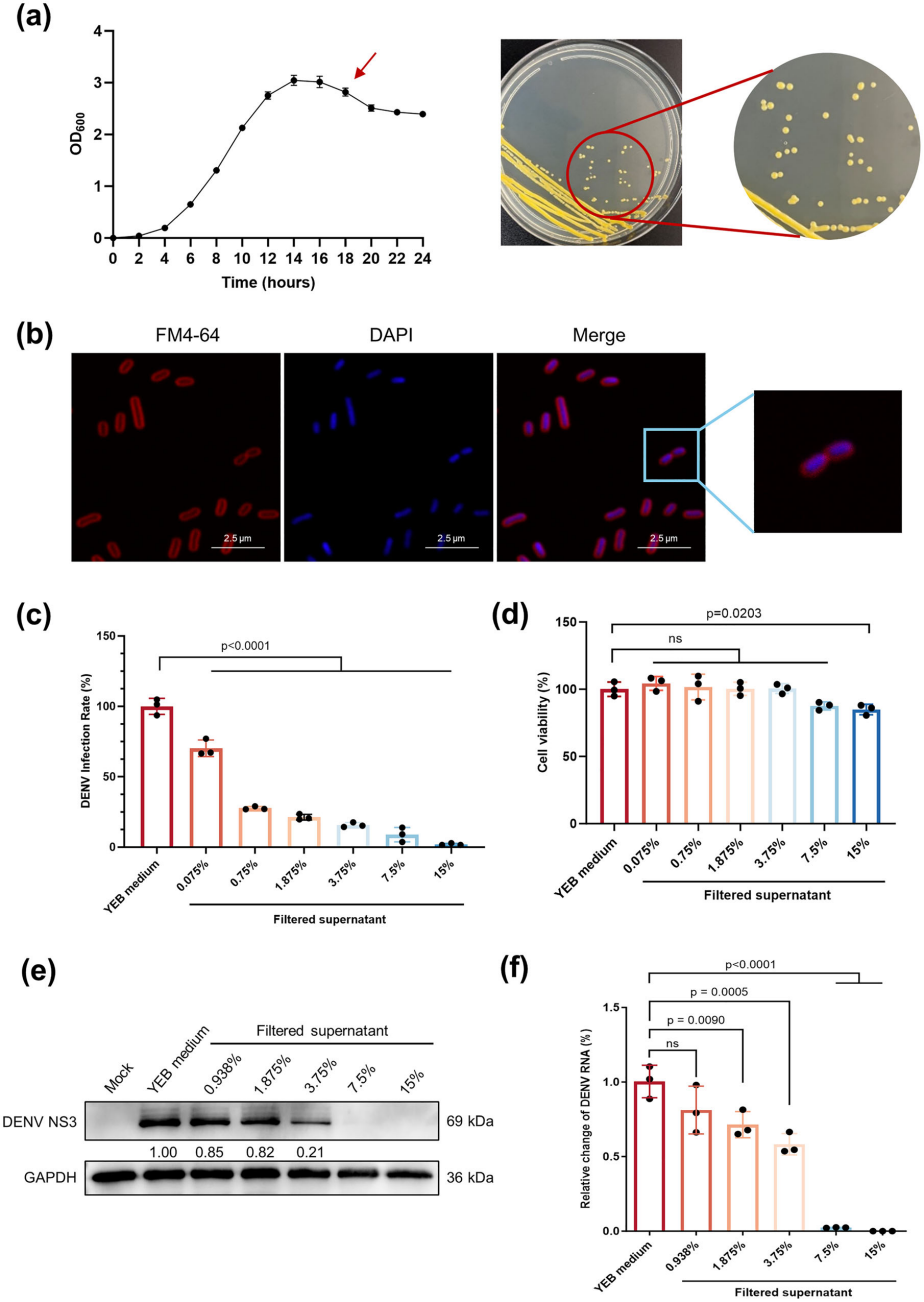
Growth, morphology of C. aquifrigidense M24 and its antiviral activity of supernatant against DENV. (a) Growth curve and colonial morphology of C. aquifrigidense M24 in YEB medium. The red arrow indicates the time point for harvesting bacteria for subsequent experiments. (b) Confocal microscopy images showing the rod‐shaped morphology and cells undergoing binary fission (indicated by enlarged figure). Bacterial membranes were stained with FM4‐64 (red), and DNA was stained with DAPI (blue). Scale bar = 2.5 µm. (c) DENV infection rate showing by different concentrations of bacterial supernatant, measured by immunofluorescence assay (one‐way ANOVA). (d) Effect of different concentrations of bacterial supernatant on the viability of cells, determined by CCK‐8 assay (one‐way ANOVA). Analysis of the inhibitory effect of different supernatant concentrations on DENV NS3 protein expression by Western blot (e) or on intracellular DENV RNA levels by qRT‐PCR (f) (one‐way ANOVA). Images were quantified by ImageJ, and the protein levels were normalized to GAPDH. Data are presented as mean ± SD (n = 3 for each group); a one‐way ANOVA test followed by Dunnett's multiple comparisons test, with the YEB medium group serving as the control group, was used in (c, d and f). ns, not significant.

To characterize the growth and morphology of *C. aquifrigidense* M24, we cultured the bacterium in YEB medium. As shown in Figure 1a, the bacterium exhibited a typical sigmoidal growth curve, with a lag phase of approximately 2 h, a logarithmic phase reaching peak density (OD_600_ = 3.05) at around 14 h, followed by a stationary phase. Single colonies were picked and subjected to at least seven generations of consecutive subculturing, during which the colony size, color and morphology on YEB solid medium showed no significant changes (Figure S1), indicating that this strain remains stable during continuous subculturing. 16S rDNA sequencing of a selected single colony further confirmed its closest phylogenetic relationship with *C. aquifrigidense*, with sequence identity > 99% (Figure S2). Gram staining showed that it is a Gram‐negative bacterium (Figure S3). Based on the growth curve, bacteria were harvested during the stationary phase (OD_600_ ≈ 2.82) for subsequent experiments. Confocal microscopy observation revealed a rod‐shaped morphology of the strain, with frequent observations of binary fission indicating it as the primary mode of reproduction (Figure 1b).

Next, we assessed the antiviral activity of the filter‐sterilized bacterial supernatant against DENV infection. Immunofluorescence assays revealed a dose‐dependent inhibition of DENV infection, with inhibition rates ranging from 29.72% (at 0.075% supernatant) to 97.98% (at 15% supernatant) when compared to the YEB medium control group (Figure 1c, Figure S4). Cytotoxicity measured by CCK‐8 assay was low at effective concentrations; cell viability remained above 95% at concentrations ≤ 3.75%, and was 84.92% at the highest concentration (15%) (Figure 1d).

Further verification by Western blot demonstrated a gradual decrease in viral NS3 protein levels with increasing supernatant concentration, while GAPDH levels were stable (Figure 1e). Correspondingly, qRT‐PCR results demonstrated that the supernatant significantly reduced intracellular viral RNA levels with increasing doses. Compared to the control group (set as 100%), treatment with 1.875%, 3.75%, 7.5% and 15% supernatant significantly reduced viral RNA levels to 71.48%, 58.30%, 2.37% and 0.01%, respectively (Figure 1f). Together, these results confirm that the bacterial supernatant contains secreted factor(s) that potently and specifically inhibit DENV infection.

### 3.2 Observation, Purification and Antiviral Activity of BEVs

**FIGURE 2:**
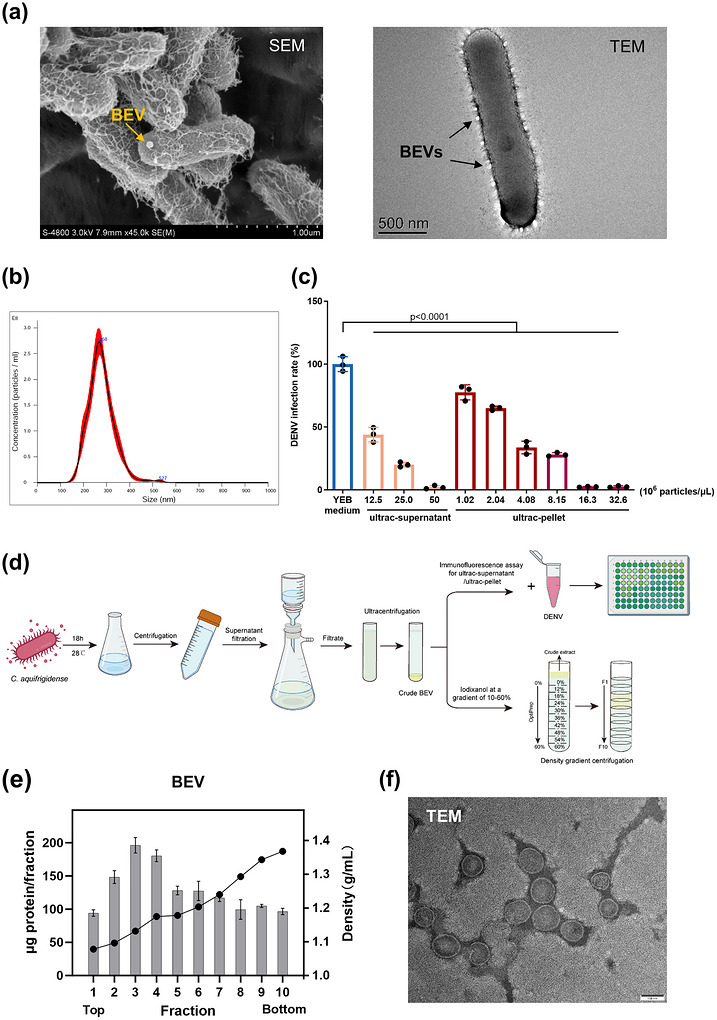
Isolation, purification and characterization of BEVs. (a) SEM (left) and TEM (right) images showing bacterial surface structures and secreted BEVs (indicated by arrows). Scale bar = 1 µm or 500 nm. (b) Characterization of BEV particle size and concentration by NTA. The sample was diluted 1:10,000 and analyzed in triplicate. Data are presented as mean ± SD. (c) Inhibitory activity of the ultracentrifugation supernatant and pellet on DENV infection, assessed by immunofluorescence assay (one‐way ANOVA). (d) Schematic diagram of BEVs isolation and purification through fractions after iodixanol density gradient centrifugation. (e) Measured density and protein content of each gradient fraction. (f) TEM image of purified BEVs after negative staining. Scale bar = 100 nm. Data are presented as mean ± SD (n = 3 for each group); a one‐way ANOVA test followed by Dunnett's multiple comparisons test, with the YEB medium group serving as the control group, was used in (c).

Electron microscopy was used to visualize bacterial ultrastructure. Scanning Electron Microscopy (SEM) showed a rough bacterial surface with numerous pili structures attached (Figure 2a, left panel). Furthermore, scattered granular structures could be observed around the bacterial cells. Transmission electron microscopy (TEM) results not only confirmed the presence of pili but also clearly revealed an abundance of membrane‐derived vesicular structures located on the bacterial surface and within the interstices of the pili (Figure 2a, right panel). Aggregates of similar vesicles were also observed in the areas surrounding the bacteria (Figure S5), indicative of active BEVs production.

To isolate these vesicles, the bacterial supernatant was subjected to ultracentrifugation. The resuspended pellet was first characterized by NTA, revealing a concentration of (3.26 ± 0.13) × 10^9^ particles/µL and an average particle size of 277.7 ± 2.0 nm (Figure 2b). Nanoscale flow cytometry analysis of DiO‐stained BEVs further confirmed the presence of high‐concentration nano‐sized vesicular particles, with the majority of vesicle particles predominantly distributed in the size range of 100–300 nm (Figure S6). To further identify the distribution of the antiviral active components, the inhibitory effects of both the ultracentrifugation supernatant and the vesicle‐containing pellet on DENV infection were evaluated by immunofluorescence assay. The results demonstrated a concentration‐dependent inhibition for both fractions. Specifically, the supernatant fraction inhibited DENV infection by 56.11%, 79.89% and 97.93% at final concentrations of 12.5, 25 and 50×10^5^ particles/µL, respectively. The pellet fraction containing the vesicles exhibited inhibition rates of 22.42%, 34.98%, 66.34%, 71.90% and 97.61% at final concentrations of 1.02, 2.04, 4.08, 8.15, 16.3, 32.6×10^6^ particles/µL, respectively (Figure 2c). No significant cytotoxicity of BEVs was observed at concentrations below 16.3 × 10^6^ particles/µL (Figure S7). The data indicate that both the supernatant and the pellet fractions inhibit DENV infection in a concentration‐dependent manner. However, the pelleted vesicle fraction demonstrated significantly greater antiviral potency.

The crude BEVs preparation was further subjected to 10%‐60% iodixanol density gradient ultracentrifugation for purification. Ten fractions were then collected sequentially from the top to the bottom of the gradient (Figure 2d). Measurement of the density and protein content of each fraction revealed that Fraction 3 (F3) and 4 (F4) exhibited the highest protein content and densities of 1.13 and 1.18 g/mL, respectively (Figure 2e). Interestingly, under a final concentration of 25%, F3 and F4 exhibited the most potent inhibition of DENV infection, with inhibition rates exceeding 85% (Figure S8), but F4 exhibited significant cytotoxicity (data not shown). Therefore, F3 was selected for further purification. TEM of the purified F3 preparation confirmed the presence of intact, bilayer BEVs with diameters ranging from 40 to 260 nm, mostly around 100 nm (Figure 2f).

### 3.3 BEVs Inhibit DENV Infection Primarily by Directly Inactivating Viral Particles

Since BEVs presented in bacterial supernatant was shown to effectively inhibit DENV infection, the precise stage(s) of the viral life cycle targeted remained unclear yet. Potential mechanisms could involve virus binding, entry, or post‐entry within the host cell. Here, we investigated which step of the DENV life cycle is targeted by BEVs.

**FIGURE 3:**
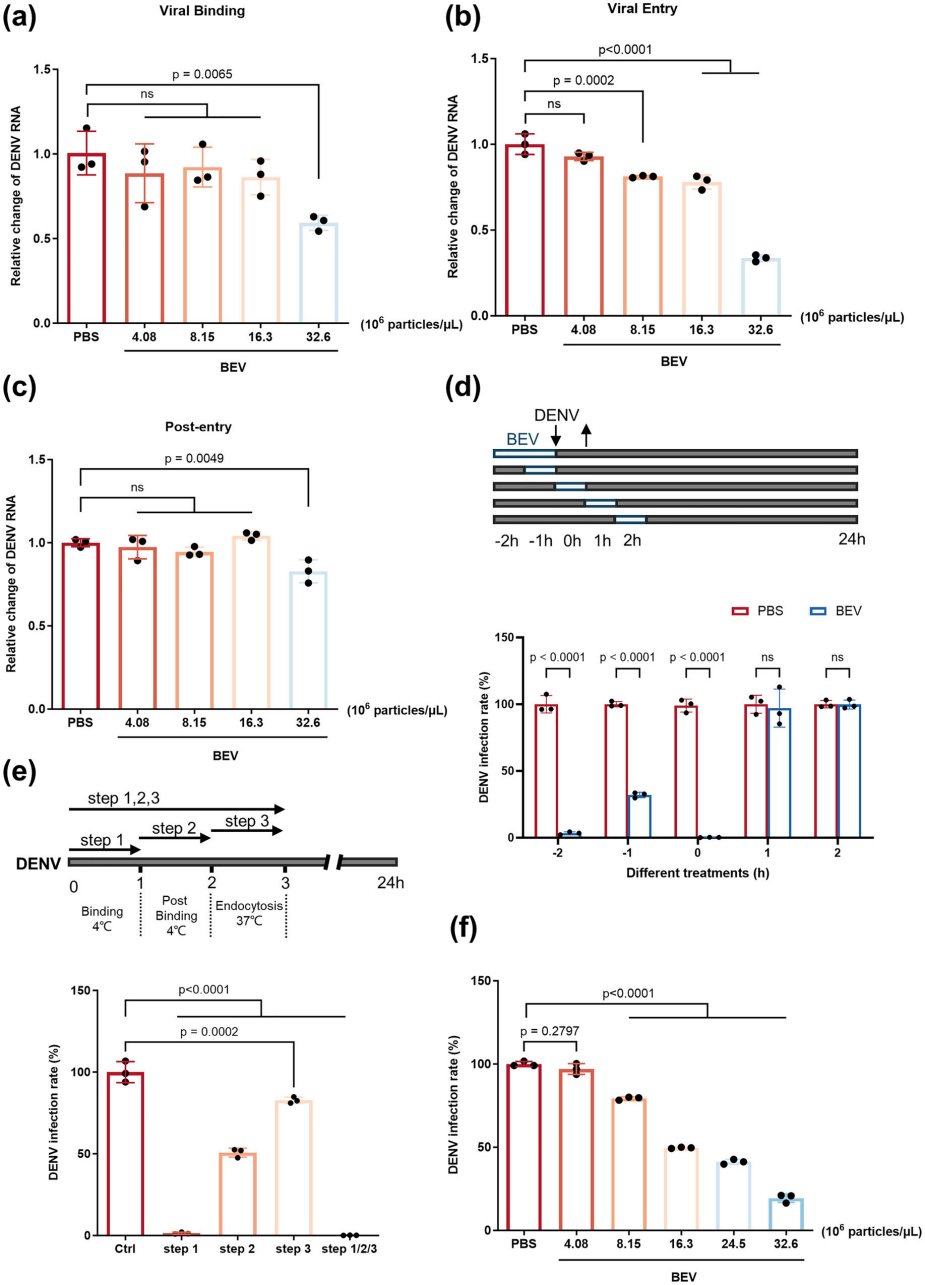
BEV‐mediated inhibition of DENV infection primarily occurs through direct targeting of viral particles. (a) Viral binding assay. (b) Viral entry assay. (c) Post‐entry assay. Viral RNA levels were assessed by qRT‐PCR and are expressed relative to the PBS control (one‐way ANOVA). (d) Effect of BEVs on viral infectivity treated at different time points was assessed (unpaired Student's t‐test). (e) Refined time‐of‐addition assay for BEVs (32.6×106 particles/µL) (one‐way ANOVA). (f) Virus pre‐incubation assay. Viral infectivity was analyzed by immunofluorescence (one‐way ANOVA). Data are presented as mean ± SD (n = 3 for each group); one‐way ANOVA test followed by Dunnett's multiple comparisons test, with the PBS group or the Ctrl group serving as the control group, was used in (a, b, c, e and f) and unpaired Student's t‐test was used in (d). ns, not significant.

For the virus binding assay, cells were co‐incubated with DENV and different concentrations of BEVs at 4°C for 1 h to allow virus attachment without internalization. Compared to the PBS control group, cell surface viral RNA levels in the 4.08, 8.15 and 16.3×10^6^ particles/µL BEVs treatment groups decreased slightly but not significantly, while the 32.6×10^6^ particles/µL BEVs treatment group showed a significant reduction to 59.34% (Figure 3a), suggesting that high concentration BEVs (32.6×10^6^ particles/µL) significantly reduced virus attachment to cells.

For the virus entry assay, after cells were bound with DENV and BEVs at 4°C for 1 h, they were transferred to 37°C for 1 h of synchronized infection. The results demonstrated that intracellular viral RNA levels in the 4.08, 8.15, 16.3 and 32.6×10^6^ particles/µL BEVs treatment groups decreased to 92.99%, 81.25%, 78.03% and 33.68% of the control group, respectively (Figure 3b), indicating that BEVs potently inhibited viral entry in a clear dose‐dependent manner.

For the post‐entry assay, cells were first infected with DENV for 1 h. After removal of non‐internalized virus, BEVs were added for 1 h of treatment, followed by continued culture in complete medium for 24 h. qRT‐PCR results showed that BEVs treatment had only a slight effect on post‐entry steps, with a decrease to 82.87%, at the concentration of 32.6×10^6^ particles/µL (Figure 3c).

Given that viral RNA levels do not fully reflect viral infectivity, we further used immunofluorescence assay to validate the effect of BEVs on the DENV entry process. Results showed that adding 32.6×10^6^ particles/µL BEVs simultaneously with the virus or 2 h before infection produced the most significant inhibitory effect. However, no inhibition was observed when BEVs were added after infection (Figure 3d), further confirming that BEVs primarily act during the early stages of viral infection. To refine the specific point of action of BEVs during early infection, we added BEVs at three different time points: virus binding (step 1), post‐binding (step 2) and endocytosis (step 3). Results demonstrated that adding BEVs during the binding step strongly inhibited DENV infection, whereas adding them at later steps gradually diminished the inhibitory effect (Figure 3e), indicating that the strongest inhibition of BEVs occurred during the virus attachment phase.

To further investigate whether BEVs directly target viral particles, we pre‐incubated DENV with different concentrations of BEVs at 37°C for 1 h before infecting cells. Immunofluorescence results demonstrated that pre‐treatment with 8.15, 16.3, 24.5, 32.6×10^6^ particles/µL BEVs reduced viral infectivity by approximately 20.6%, 50.34%, 58.69% and 80.57%, respectively (Figure 3f), indicating that pre‐incubating DENV with BEVs before infection directly reduced viral infectivity in a concentration‐dependent manner. In summary, BEVs significantly reduce viral infectivity primarily by directly interacting with and inactivating viral particles, with a certain inhibitory effect on the cellular entry step.

### 3.4 BEV‐DENV Interaction Abolishes Infectivity and Alters Viral Component Distribution

**FIGURE 4:**
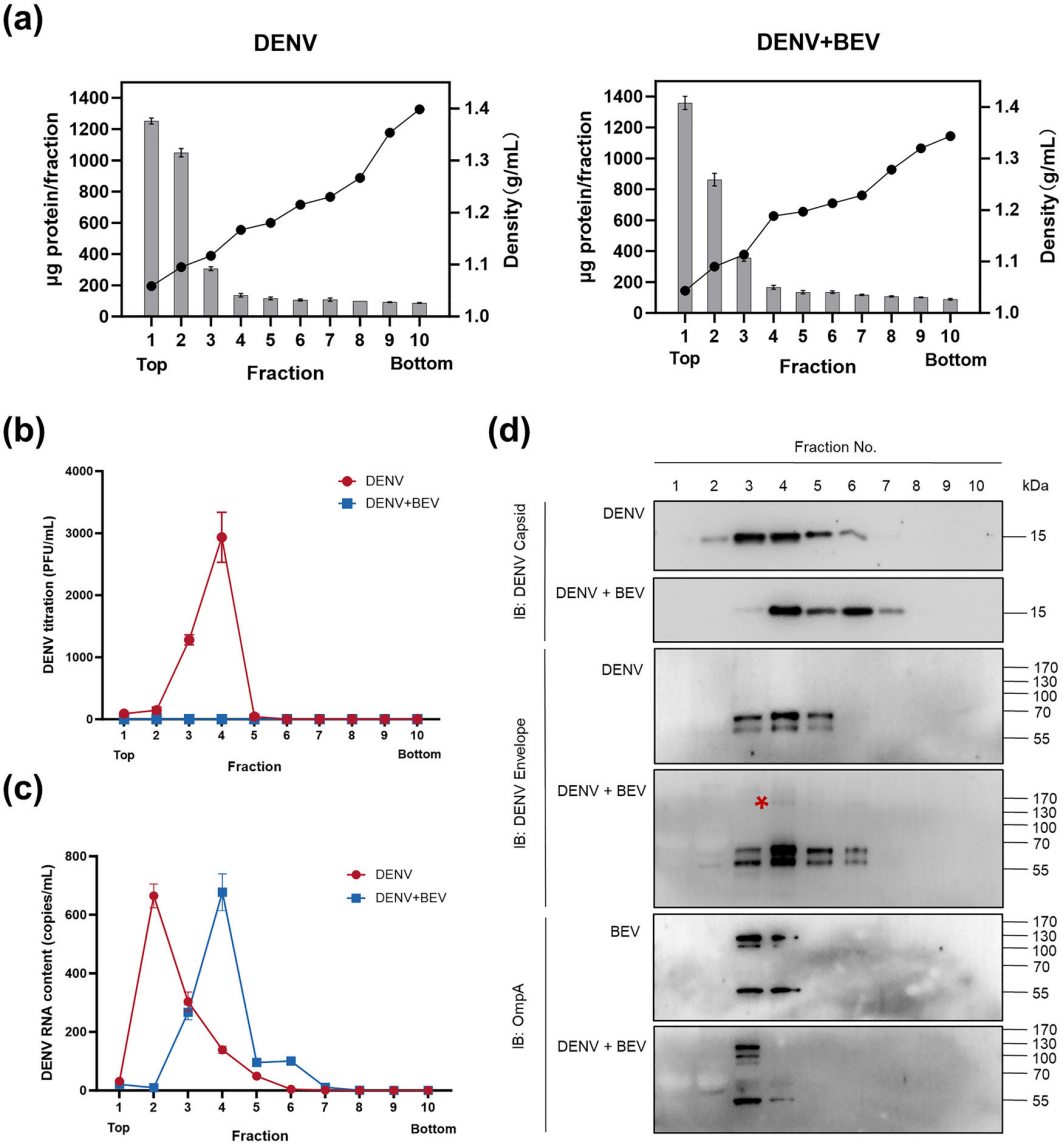
BEV‐DENV interaction leads to viral structural disassembly and loss of infectivity. (a) Total protein content and density distribution of fractions after density gradient centrifugation. (b) Viral titer measurement of fractions showing that BEVs treatment completely abolished DENV infectivity. (c) qRT‐PCR analysis showing the distribution changes of DENV genomic RNA across fractions. Data are presented as mean ± SD (n = 3 for each group). (d) Western blot analysis of the distribution of viral Capsid (C), Envelope (E) and OmpA proteins across fractions. The asterisk indicates possible E protein dimers or trimers appearing in the BEV‐treated group.

To further elucidate the interaction between BEVs and DENV, we performed an *in vitro* co‐incubation assay followed by density gradient ultracentrifugation. The density and protein content of ten fractions collected sequentially from the top of the gradient were analyzed. The results were shown in Figure 4a. Compared to the DENV‐alone group, the BEV‐DENV mixture group exhibited a significant decrease in total protein content specifically in fraction 2 (from 1049.23 µg/mL to 863.01 µg/mL), while total protein content increased in most other fractions (Figure 4a). Regarding density distribution, compared to fractions 4 and 5 (densities of 1.17 and 1.18 g/mL, respectively), where the virus primarily banded in the DENV‐alone group, the corresponding F4 and F5 in the BEV‐DENV mixture group showed increased densities (1.19 and 1.20 g/mL, respectively). Conversely, the density of F3 slightly decreased from 1.12 g/mL to 1.11 g/mL (Figure 4a). The protein content and density profiles of the fractions suggest that certain changes may have occurred between BEVs and DENV.

Critically, while infectious virus was detected in multiple fractions (F1 to F5) from the DENV‐alone control, no infectivity was observed from any fraction of the BEV‐DENV mixture (Figure 4b), demonstrating a complete loss of infectivity upon interaction.

Given that BEVs treatment abolishes DENV infectivity, viral components were further analyzed for the viral genomic RNA and structural proteins across the fractions. The quantification results of DENV RNA are shown in Figure 4c. In the DENV‐alone control group, DENV RNA was primarily detected in fractions F2‐F5, with the highest level in F2. In contrast, for the BEV‐DENV mixture group, the highest RNA content was found in F4, which exhibited an approximately 3.87‐fold increase compared to the corresponding control fraction. Furthermore, the RNA levels in F5 and F6 were significantly higher than those in the control group, particularly in F6, where the RNA content increased about 23‐fold. However, almost no viral RNA was detected in F2 (Figure 4c).

Western blot analysis for the structural capsid (C) and envelope (E) proteins yielded complementary results (Figure 4d). In the DENV‐alone control, the C protein was detectable in fractions F1‐F7, with the strongest signals in F3‐F5. For the BEV‐DENV mixture, the C protein was also present in F3‐F7, but its distribution peaked in F4 and F6. Similarly, the E protein in the control was mainly localized to F3‐F5. However, in the BEV‐DENV mixture, the E protein was detected across a broader range (F2‐F7), primarily in F3‐F6. Intriguingly, the E protein in the mixture, particularly in F4, displayed suspected dimeric (or multimeric) forms (Figure 4d, bands were labeled with *), suggesting BEVs induce conformational changes or aggregation of the E protein. Furthermore, the distribution of OmpA protein (used as an BEV marker protein) was also analyzed for the fractions. Compared to the BEV‐alone group (where OmpA was mainly enriched in F3 and F4), the OmpA protein content in the BEV‐DENV mixture was significantly reduced in F4, with the high molecular weight band almost completely disappearing and abnormal OmpA bands appeared in F3 (Figure 4d), indicating BEV membrane integrity was compromised. Collectively, these findings demonstrate that BEVs interact with DENV, disrupting the viral envelope, increasing particle density, inducing protein alterations and ultimately leading to a loss of infectivity.

### 3.5 DENV‐BEV Interaction Alters Lipid Distribution in Gradient Fractions

**FIGURE 5:**
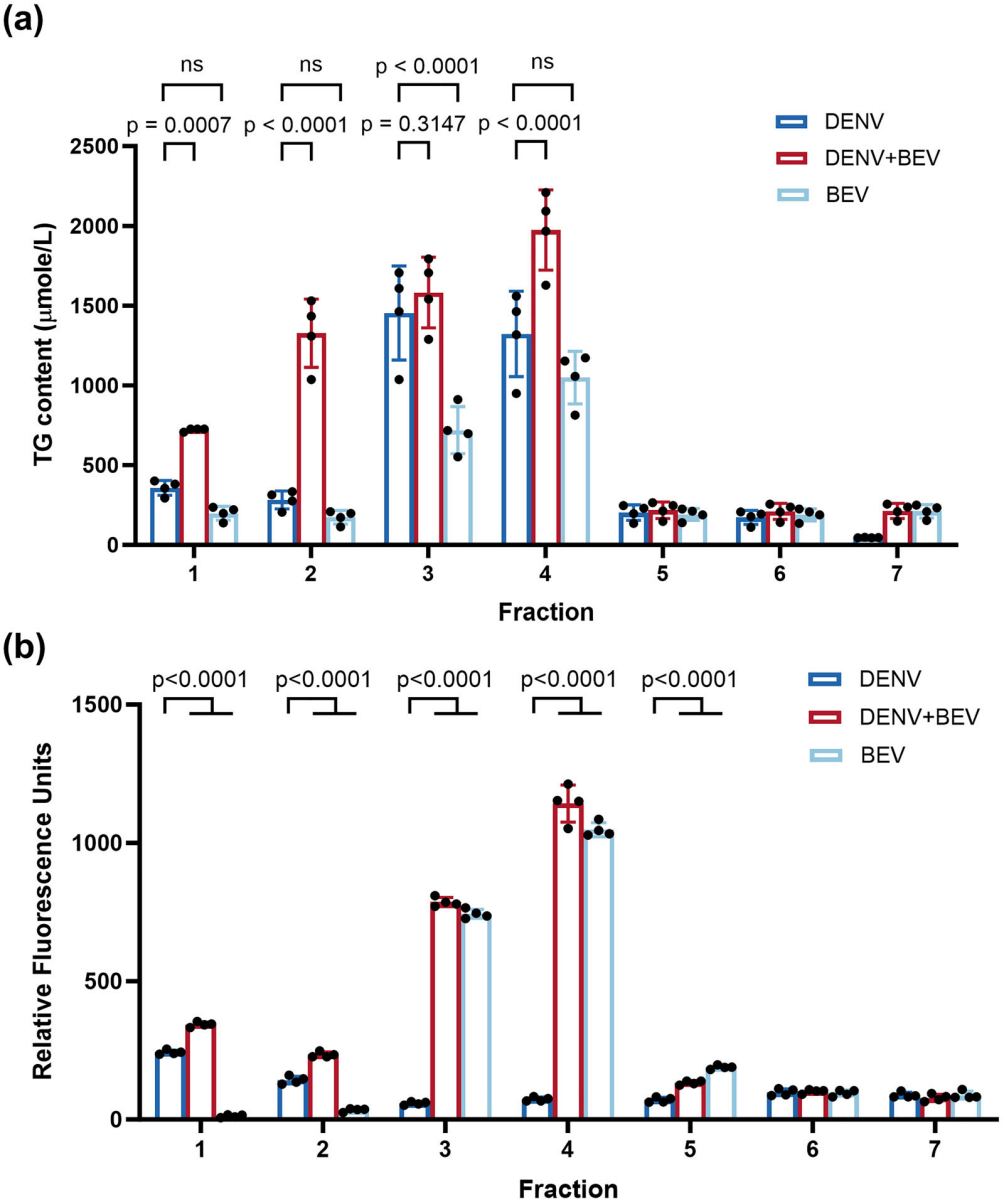
BEV treatment increases triglyceride (TG) content and total membrane lipid in low‐density fractions. Quantitative measurement of TG content (a) and total membrane lipids (b) in density gradient fractions. Data are presented as mean ± SD (n = 3 for each group); one‐way ANOVA test followed by Dunnett's multiple comparisons test, with the DENV group serving as the control group, was used in (a and b). ns, not significant.

To explore the potential mechanism behind the BEV‐induced density shift of viral particles, we quantified lipid changes in gradient fractions F1‐F7. As shown in Figure 5a, in both the DENV‐alone and BEV‐alone ultracentrifugation groups, the highest triglyceride (TG) content was found in F3 and F4, with values of 1453.49 and 1322.67 µmol/L, and 719.48 and 1048.93 µmol/L, respectively (Figure 5). After BEV treatment, the TG content increased sharply in the low‐density fractions (F1, F2) to 721.90 and 1327.52 µmol/L, which were 2.02‐fold and 4.71‐fold higher than in the DENV‐alone group, and 3.64‐fold and 7.65‐fold higher than in the BEV‐alone group, although the peak TG content remained in F3 and F4 (1581.88 and 1974.32 µmol/L, respectively) (Figure 5a).

To further determine changes in total membrane lipids across fractions containing viral particles and BEVs, we performed relative quantification using the lipophilic fluorescent probe FM4‐64. This probe exhibits very weak fluorescence in aqueous environments but shows a significant increase in fluorescence intensity upon incorporation into hydrophobic membrane environments. The results of relative fluorescence units showed that in the BEV‐alone group, the highest membrane lipid content was in F4 and F3, with fluorescence intensity values of 1047.75 and 743.5, respectively (Figure 5b), consistent with the previously observed distribution of the antiviral active fractions and the main localization of BEVs. In the DENV‐alone group, the highest membrane lipid content was in F1 and F2, with fluorescence intensity values of 243 and 142.5, respectively, rather than in the fractions (F3 and F4) with the highest viral infectivity, suggesting the possible presence of non‐infectious membranous materials in the virus preparation. After BEVs treatment, the highest membrane lipid content remained in F4 and F3 (1142 and 786, respectively). Compared to the sum of the fluorescence intensities of the corresponding fractions in the BEV‐alone and DENV‐alone groups, these two fractions did not show a significant increase (0.98‐fold and 1.02‐fold). However, it is noteworthy that the membrane lipid content in F1 and F2 of the BEV‐DENV mixture increased (343.25 and 234, respectively), which was 1.34‐fold and 1.32‐fold higher than the sum of the fluorescence intensities in the corresponding fractions of the individual groups (Figure 5b). These data suggest that BEV treatment disrupts the viral envelope, leading to a redistribution and alteration of its lipid composition.

### 3.6 BEVs Cause Direct Structural Damage to DENV Virions

**FIGURE 6:**
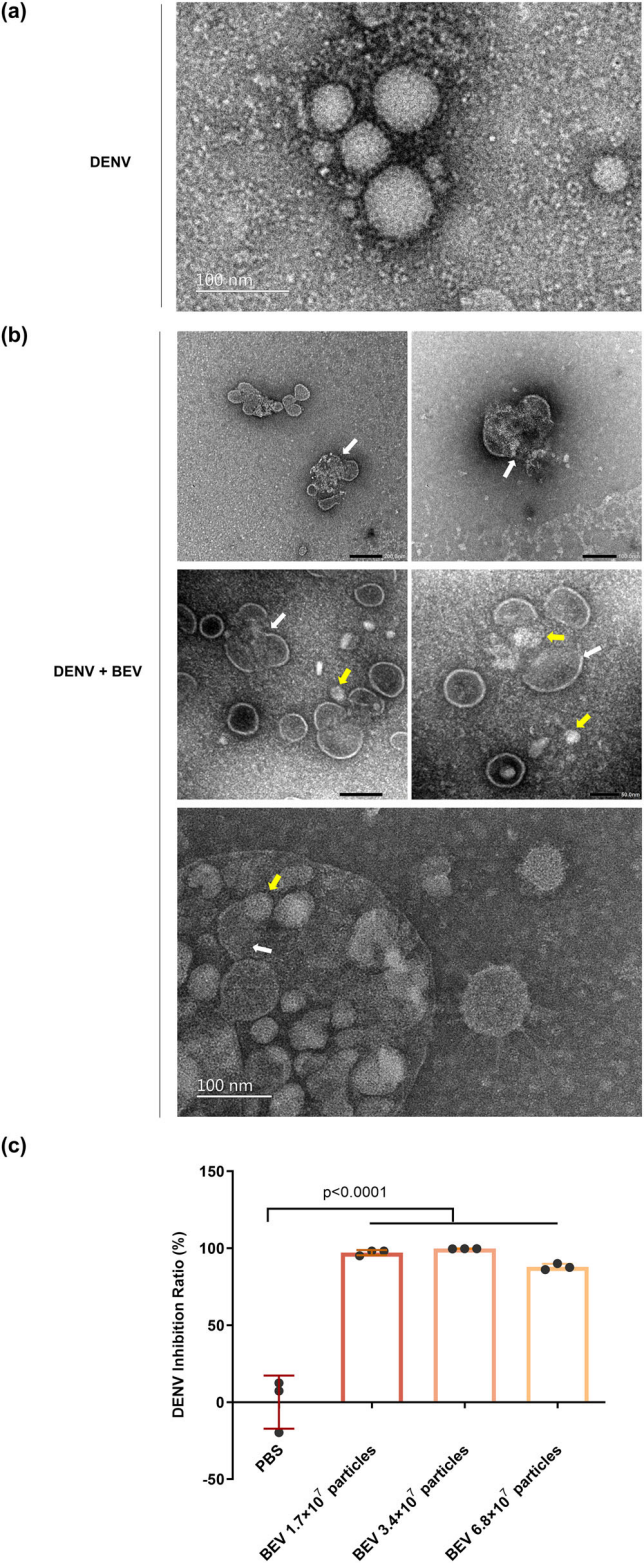
Direct observation of structural damage to DENV following interaction with BEVs by TEM and viral RNA exposure assay. (a) Untreated DENV particles, showing intact spherical structures. Scale bar = 100 nm. (b) Morphology of DENV after co‐incubation with BEVs under different conditions: high‐dose BEVs (top row), medium‐dose BEVs (middle row) and low‐dose BEVs plus high‐dose DENV (bottom row). BEV aggregation and lysis are indicated by white arrows, and irregular electron‐dense particles by yellow arrows. Scale bars are shown in each image. (c) BEV treatment compromises virion structural integrity, leading to viral RNA exposure. Following incubation of DENV with different concentrations of BEVs, the samples were treated with RNase A, and the remaining undegraded viral RNA was quantified by qRT‐PCR. Data are presented as mean ± SD (n = 3 per group). Statistical analysis was performed using one‐way ANOVA followed by Dunnett's multiple comparisons test, with the PBS group as the control.

To directly test the hypothesis that BEV treatment leads to viral structural damage, we employed TEM to visualize the morphological characteristics of DENV alone and after mixing with BEVs. As shown in Figure 6a, untreated DENV appeared as smooth, spherical particles, with diameters ranging from 40 to 60 nm.

After co‐incubation of DENV (∼1.4×10^4^ PFU) with a higher dose (6.8 × 10^7^ particles) of purified BEVs, intact viral particles were rarely observable; only small aggregates of BEVs were visible, with the majority of these vesicles undergoing lysis (Figure 6b, top row). When the BEV dose was reduced to 3.4 × 10^7^ particles, intact viral particles remained undetectable, but more BEVs with relatively intact morphology were observed, alongside vesicles in the process of disintegration. Furthermore, numerous smaller, irregularly shaped particulate materials were visible in the field of view (Figure 6b, middle row). To further characterize the interaction between them, we adjusted the mixing ratio by reducing the BEV dose to 1.7 × 10^7^ particles while increasing the DENV amount to 5.6 × 10^4^ PFU. Under this condition, more vesicles and viruses undergoing morphological changes were observed (Figure 6b, bottom row). These observations confirm that BEVs interact with the virus in a dose‐dependent manner, disrupting the integrity of the viral envelope and leading to irreversible structural disintegration.

To test the hypothesis that BEVs destroy the membrane integrity of the virion, we performed a viral RNA exposure assay. Different concentrations of BEVs were mixed with 1.4 × 10^4^ PFU of DENV for 2 h at 37°C, followed by RNase A treatment to assess viral RNA degradation, which would occur if the envelope were compromised. As shown in Figure 6c, compared with the mock treatment (viruses incubated with PBS), a significant reduction in viral RNA was detected by qRT‐PCR upon BEV treatment, indicating that BEVs disrupted the membrane integrity of the virion and consequently released the viral genome. These results were consistent with the TEM observations, further supporting the virucidal activity of BEVs.

### 3.7 BEVs Exert Antiviral Effects via Membrane Fusion With the Viral Envelope

**FIGURE 7:**
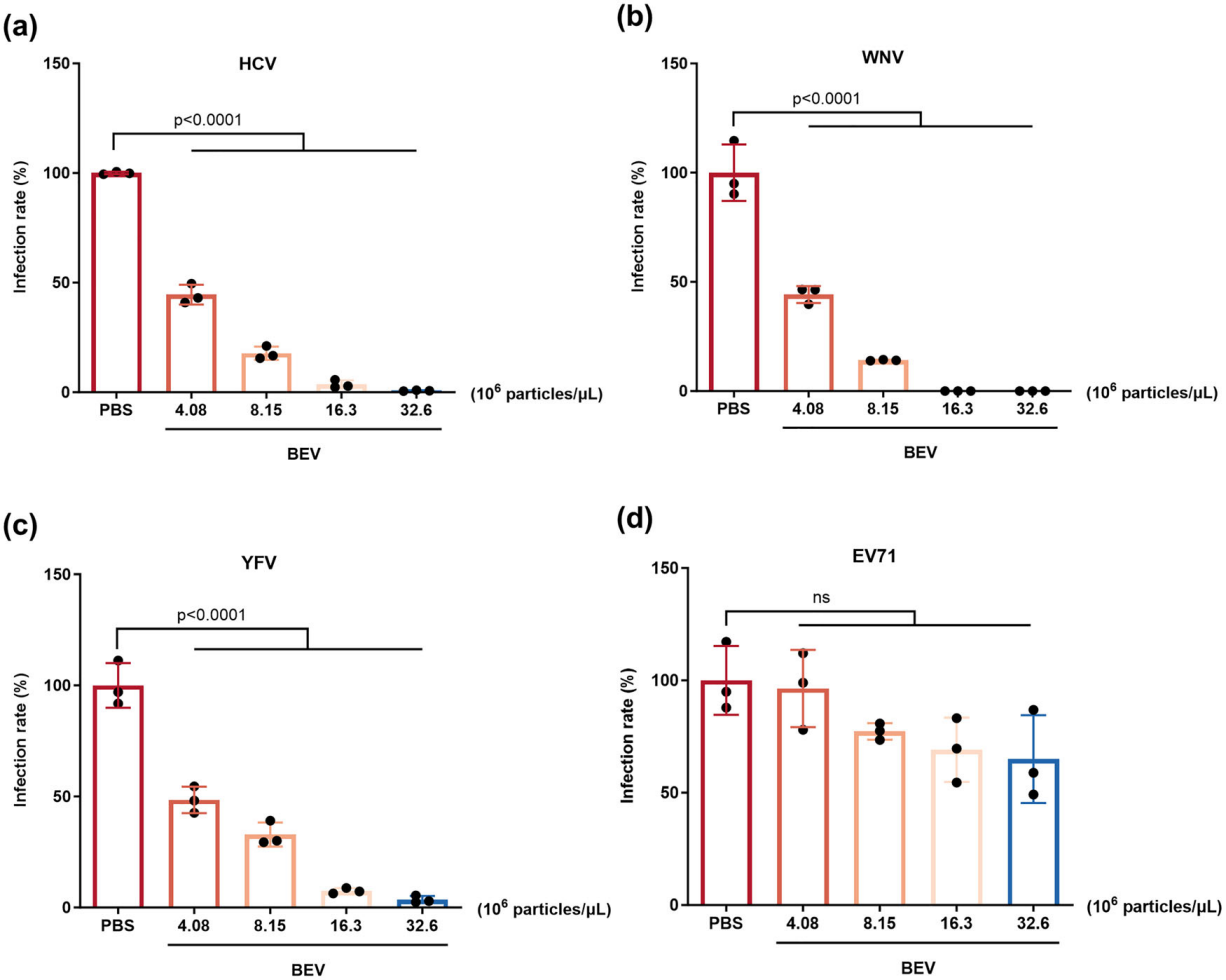
BEVs dose‐dependently inhibit enveloped viruses but not non‐enveloped EV71. (a‐d) Huh7 or RD cells were infected with HCV (a), WNV (b), YFV (c), or EV71 (d) (MOI = 1) for 1 h in the presence or absence of the indicated concentrations of BEVs (4.08‐32.6×10^6^ particles/µL). PBS served as the negative control. After 24 h, infection rates were quantified by immunofluorescence assay and are expressed as a percentage of the PBS control group. Data are presented as mean ± SD (n = 3 for each group); one‐way ANOVA test followed by Dunnett's multiple comparisons test, with the PBS group serving as the control group. ns, not significant.

To test the hypothesis that the possible target of BEVs is the viral envelope, we examined its effects on the infectivity of enveloped and non‐enveloped viruses. The results showed that, similar to its dose‐dependent inhibition of DENV, BEVs also exhibited good dose‐dependent inhibition against several enveloped viruses (e.g., HCV, WNV, YFV), but had no significant inhibitory effect on the non‐enveloped Enterovirus 71 (EV71) (Figure 7), suggesting that its antiviral effect may involve an interaction with the viral envelope.

**FIGURE 8:**
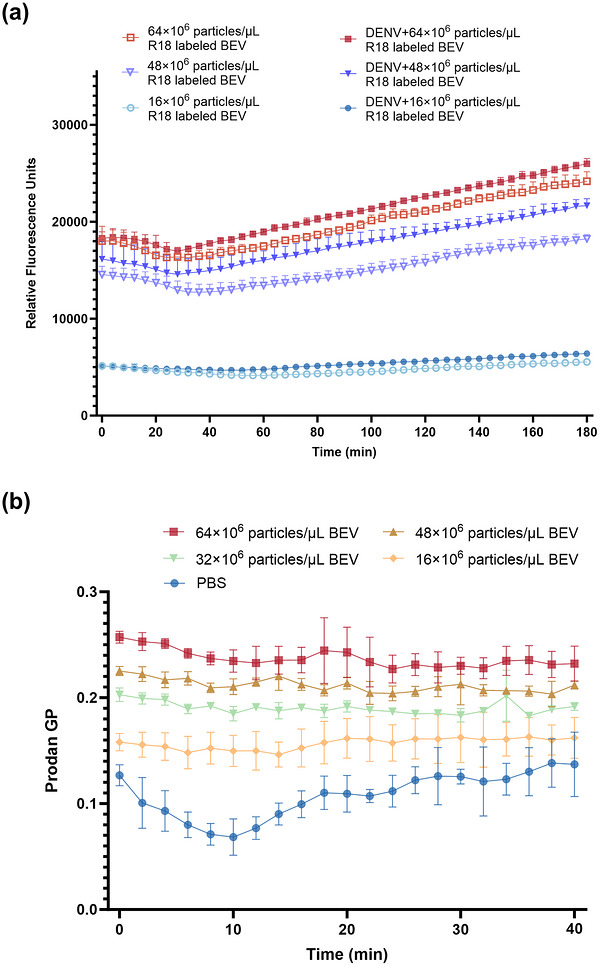
BEV treatment triggers membrane fusion. (a) Fusion kinetics of DENV mixed with the indicated concentrations of R18‐labeled BEVs. (b) Effect of BEVs on membrane fluidity, measured by the fluorescence of Prodan in a cell‐free system. Data are presented as mean ± SD (n = 3 for each group).

To further investigate the molecular mechanism by which BEVs suppress DENV infectivity, we labeled BEVs with R18 to monitor membrane fusion with DENV dynamically. R18 exhibits fluorescence self‐quenching at high concentrations; fusion between labeled BEVs and the viral envelope leads to dye dilution, dequenching and fluorescence recovery. As shown in Figure 8a, incubating different concentrations (64, 48, 16×10^6^ particles/µL) of R18‐labeled BEVs with DENV (1.4×10^4^ PFU) resulted in a concentration‐dependent increase in fluorescence, indicating dye dequenching due to lipid mixing and membrane fusion. In the BEV‐only control group, fluorescence also increased over time (Figure 8a), suggesting that BEVs may undergo self‐fusion at 37°C.

Next, the membrane dye Prodan was used to assess changes in membrane fluidity. DENV was labeled with 5 µM Prodan, mixed with different concentrations of BEVs and changes in emission spectra at 440 and 490 nm were monitored dynamically. Results showed that, compared with the PBS control, the generalized polarization (GP) values of Prodan‐labeled DENV treated with 64, 48, 32 and 16×10^6^ particles/µL BEVs were significantly increased in a dose‐dependent manner (Figure 8b). This indicates rapid lipid mixing between DENV and BEVs, resulting in a blue shift of the fluorescence spectrum and decreased membrane fluidity, further supporting the occurrence of membrane fusion.

## 4 Discussion

This study presents the first comprehensive evidence that BEVs derived from *C. aquifrigidense* M24 effectively inhibit DENV infection. We demonstrate that the underlying mechanism involves membrane fusion between BEVs and the viral envelope, leading to irreversible structural disintegration and functional inactivation of viral particles in the extracellular environment, accompanied by reduced membrane fluidity and lipid rearrangement.

Bacterial membrane vesicles, with their diverse biogenesis pathways and compositions, play various roles in different processes of bacteria. These include supporting bacterial growth (e.g., exporting bioactive molecules, DNA transfer and waste disposal) and mediating host interactions (e.g., delivering virulence factors, inducing immunomodulation) (Kulkarni and Jagannadham 2014). Recent studies also highlight their role as a defense mechanism against phages. For instance, Tamara Reyes‐Robles et al. reported that OMVs secreted by *Vibrio cholerae* competitively inhibit phage binding to bacterial surface receptors by interacting with phage tail fibers, thereby neutralizing diverse phages (Reyes‐Robles et al. 2018). However, the effect of OMVs or BEVs on human viruses, particularly mosquito‐borne viruses, has not been previously reported.

Here, we systematically evaluated the effect of *C. aquifrigidense* M24 supernatant and BEVs on the DENV life cycle. Both significantly suppressed DENV infectivity, viral protein expression and RNA replication dose‐dependently. While BEVs showed limited effect on viral binding or post‐entry steps at the RNA level, infectivity assays revealed that BEVs strongly inhibit infection when present during early stages—simultaneously with DENV, 2 h before infection, or during virus attachment. Crucially, pre‐incubating DENV with BEVs directly reduces infectivity. This indicates that BEVs act primarily by disrupting viral structural integrity, particularly the envelope.

To confirm structural damage as the cause of infectivity loss, we employed density gradient analysis. BEVs treatment not only abolished infectivity but also shifted the distribution of DENV RNA, capsid (C) and envelope (E) proteins toward higher‐density fractions. In the untreated control, C and E proteins were primarily distributed in low‐density fractions (F3, F4; densities ∼1.12 and 1.17 g/mL, respectively). Following BEVs treatment, these proteins shifted towards higher‐density fractions (F4‐F7; densities ∼1.19, 1.20, 1.21 and 1.23 g/mL). Detection of viral genomic RNA corroborated this shift, showing a redistribution from the primary fraction (F2) in the control to the higher‐density fraction (F4) after BEVs treatment. This suggests envelope disruption and nucleocapsid exposure, preventing proper viral entry and subsequent replication.

Structural alterations in the viral envelope are often accompanied by changes in its lipid composition. Lipid analysis revealed a marked increase in triglycerides and total membrane lipids in low‐density fractions after BEV–DENV interaction, indicating envelope dissociation and lipid redistribution. Concurrently, the BEV marker OmpA showed altered distribution in key fractions (F3, F4), reflecting BEV structural compromise during its interaction with the virus.

It is reported that the lipid content of flaviviruses, including DENV, is approximately 17%, with a theoretical buoyant density of about 1.30 g/mL, while experimentally measured densities typically range between 1.19‐1.24 g/mL (Kuhn et al. 2002; Schalich et al. 1996; Xie et al. 2025). In our study, infectious DENV primarily distributed in the 1.06‐1.18 g/mL range, peaking in the 1.12‐1.17 g/mL fractions, values slightly lower than those commonly reported, which could be attributed to the gradient medium used. Studies indicate that sucrose gradient centrifugation yields relatively lower density values, cesium chloride gradients yield higher values and the iodixanol medium used here typically results in even lower buoyant densities than sucrose gradients. This reasonably explains the density distribution profile observed in our study. During DENV maturation, the E protein on the particle surface undergoes dynamic oligomeric state transitions: from prM‐E heterodimers forming trimeric spikes on immature viruses, to E protein homodimers on mature viruses and finally to trimeric forms in the post‐fusion state (Hsieh et al. 2014; Rani et al. 2024; Zhang et al. 2012). In this study, we also observed suspected E protein dimers and aggregates in BEV‐treated samples, suggesting that BEVs may induce conformational rearrangements resembling those during viral maturation or fusion.

Further key evidence supporting direct morphological disruption of DENV by *C. aquifrigidense* M24 BEVs comes from TEM. Untreated DENV particles were smooth and spherical, consistent with reported morphological characteristics of mature DENV (Barreto‐Vieira et al. 2020; Caldas et al. 2022; Kuhn et al. 2002). Additionally, some smaller viral particles were also observed, possibly corresponding to the subviral particles frequently produced in flavivirus‐infected cells (Barth 1999; Wang et al. 2009; Welsch et al. 2009). Moreover, morphology of DENV virion is closely related to maturity and environmental temperature (X. Z. Zhang et al. 2013). Under cryo‐EM, mature DENV appears smooth and about 50 nm in diameter, while immature viruses, with their surface spikes, have diameters increased to about 60 nm. Furthermore, viral conformation is temperature‐dependent: virus prepared at 28°C (e.g., mosquito host temperature, as used in this study) primarily exhibits a smooth morphology; whereas at 37°C (human host temperature), virion diameter increases with exposed membranes, presenting an intermediate conformation.

Although we could clearly observe the morphology of untreated DENV, capturing the continuous structural changes induced by BEV treatment proved highly challenging. This is likely due to the highly efficient and rapid nature of the BEV‐DENV interaction, meanwhile it is critically dependent on their concentration ratio. After BEV treatment, intact virions were rare, with prevalent BEV aggregation, lysis and small irregular particles—likely nucleocapsids or BEV debris. This is a limitation of conventional TEM analysis performed without immunogold labeling targeting the capsid (C) protein. It is reported that the flavivirus nucleocapsid (composed of C protein and viral RNA) resides inside the lipid envelope and is about 21–27 nm in diameter (Cheng et al. 1995; Kuhn et al. 2002). These observations strongly support BEV‐induced envelope disruption. The absence of BEV activity against non‐enveloped EV71 further confirms that the mechanism depends on the viral envelope.

For enveloped viruses, membrane fusion is a key evolutionary strategy for cell entry, a process dependent on viral surface glycoproteins, such as the DENV E protein (Villalaín 2022, 2025; W. Zhang et al. 2013). Traditional fusion inhibitors (e.g., T20 targeting HIV gp41) block fusion by ‘locking’ the fusion protein in its pre‐fusion conformation. Although direct visualization of membrane fusion between BEV and DENV was not achieved, fluorescence‐based fusion and membrane fluidity assays support the occurrence of lipid mixing. We propose that BEVs may actively ‘trigger’ premature viral fusion in a non‐cellular context, coupled with rapid BEV lysis—a mechanism distinct from traditional fusion inhibition.

This direct virus inactivation mechanism of BEVs offers two potential advantages: (1) Broad‐spectrum potential, as it targets the conserved viral fusion mechanisms; we have confirmed activity against multiple enveloped viruses like HCV, WNV and YFV. (2) Rapid action, suitable for prophylactic or topical use (e.g., intranasal/throat sprays for influenza prevention, environmental disinfectants).

Despite the promise of OMVs or BEVs in antibacterial therapy and Nano vehicles‐based drug delivery, challenges remain: yield and composition are influenced by bacterial growth conditions (Toyofuku et al. 2019); safety profiles require further evaluation in more complex infection models due to the complex components of cargo (Rumbo et al. 2011); *in vivo* effective delivery to infection sites must be optimized (Lei et al. 2024; M. Li et al. 2020; Tamburini et al. 2023); and the potential immunogenicity (innate immune activation) and toxicity risk of BEVs or OMVs derived from environmental bacteria require *in vivo* evaluation when administered systemically. Future work should also explore combinations of OMVs or BEVs with antiviral drugs to enhance efficacy and prevent resistance.

This study demonstrates that *C. aquifrigidense* M24 BEVs inhibit DENV infection through a novel ‘fusion‐triggering structural disruption’ mechanism. This process involves membrane fusion between BEVs and the viral envelope, leading to reduced membrane fluidity, irreversible lipid rearrangement, structural disintegration and ultimately, loss of viral infectivity. These BEVs also directly inactivate other enveloped viruses (e.g., HCV, WNV, YFV) *in vitro*. Different from traditional strategies that inhibit intracellular fusion or replication, this mechanism appears to involve irreversible, extracellular inactivation based on viral structural disruption, which may offer unique benefits, including potential physical efficacy and lower susceptibility to escape mutations. Future studies should identify the specific active component(s) on BEVs responsible for the antiviral effect, elucidate their molecular interactions with viral fusion proteins, and evaluate the safety and efficacy of BEVs in more advanced models for topical and combination therapies.

## Author Contributions

**Yaqi Gao**: Investigation, software, data curation, visualization, writing – original draft. **Lijian Zhang**: Software, formal analysis, writing – original draft, data curation. **Tianci Zhang**: Investigation, software, data curation, formal analysis. **Qiufeng Yao**: Investigation, methodology. **Ruifang Gao**: Software, methodology. **Yue Wang**: Supervision, conceptualization. **Yunpeng Zhao**: Resources. **Tingting Zhou**: Supervision, investigation. **Jikuai Chen**: Software, methodology. **Xing Zhang**: Software, methodology. **Hao Ren**: Resources. **Yongzhe Zhu**: Resources. **Ping Zhao**: Supervision, project administration, resources. **Zhongtian Qi**: Supervision. **Li Luo**: Formal analysis, writing – review and editing, supervision, funding acquisition, conceptualization. **Zhaoling Qin**: writing – original draft, writing – review and editing, supervision, methodology, funding acquisition, data curation, conceptualization.

## Funding

We acknowledge funding from Shanghai University Interdisciplinary Collaborative Project (LH2025035), Naval Medical University 2023 Innovation Capability Development Fund for undergraduate and Naval Medical University 2024 Cultivation Project of Basic Medical Research (2024MS003).

## Conflicts of Interest

A patent has been filed as 202511858112.5 by Dr. Zhaoling Qin entitled ‘A functional outer membrane vesicle and its preparation and application’. All other authors declare that they have no competing interests.

## Supporting information




**Supplementary Materials**: jev270302‐sup‐0001‐SuppMat.docx

## Data Availability

The data that support the findings of this study are available from the corresponding author upon reasonable request.
