# Supplementary material for: Bacterial Extracellular Vesicles (BEVs) Derived from Chryseobacterium Inhibit Dengue Virus Infection by Disrupting Its Structural Integrity
Source: J Extracell Vesicles. 2026 Jun 23;15(6):e70302. doi: 10.1002/jev2.70302 (PMC13291215; doi:10.1002/jev2.70302)
Supplement: Supplementary file 1 — Supplementary Materials: jev270302‐sup‐0001‐SuppMat.docx [file JEV2-15-e70302-s001.docx]

Supplementary Information

Bacterial Extracellular Vesicles (BEVs) Derived from *Chryseobacterium* Inhibit Dengue Virus Infection by Disrupting its Structural Integrity

Yaqi Gao^1†^ ∣ Lijian Zhang^1†^ ∣ Tianci Zhang^1,2†^ ∣ Qiufeng Yao^1^ ∣ Ruifang Gao^3^ ∣ Yue Wang^3^ ∣ Yunpeng Zhao^3^ ∣ Tingting Zhou^4^ ∣ Jikuai Chen^5^ ∣ Xing Zhang^5^ ∣ Hao Ren^1^ ∣ Yongzhe Zhu^1^ ∣ Ping Zhao^1^ ∣ Zhongtian Qi ^1^ ∣ Li Luo^2*^ ∣ Zhaoling Qin^1*^

*^1^Department of Microbiology, Shanghai Key Laboratory of Medical Biodefense, Faculty of Naval Medicine, Naval Medical University, 800 Xiang-Yin Road, Shanghai, 200433, China* ∣ *^2^Shanghai Key Laboratory of Bio-energy Crops, Center of Plant Science, School of Life Sciences, Shanghai University, Shanghai, 200444, China* ∣ *^3^Department of Stem Cell and Regeneration Medicine, Translational Medicine Research Center, Naval Medical University, Shanghai, 200433, China* ∣ *^4^Department of Pharmaceutical Analysis, School of Pharmacy, Naval Medical University, No. 325 Guohe Road, Shanghai, 200433, China* ∣ *^5^Department of Health Toxicology, Faculty of Naval Medicine, Naval Medical University, Shanghai 200433, China*

**Correspondence:** Li Luo ([liluo@shu.edu.cn](mailto:liluo@shu.edu.cn)) ∣ Zhaoling Qin ([zhlingqin@smmu.edu.cn](mailto:zhlingqin@smmu.edu.cn))

^†^ These authors contributed equally to this work.

Keywords: Bacterial extracellular vesicles ∣ Dengue virus ∣ Antiviral mechanism ∣ Enveloped virus ∣ *Chryseobacterium*

**Materials and Methods**

#### Viruses propagation and infection assay

Cell-culture derived Hepatitis C virus particle (HCVcc) were generated by the plasmids pJFH-1 (genotype 2a, kindly provided by Dr Takaji Wakita, National Institute of Infectious Diseases, Tokyo and L. Janet Milton, Apath, Brooklyn, NY, USA), and propagated in human hepatoma Huh7.5.1 cells at 37°C. West Nile virus (WNV) and Yellow fever virus (YFV) vaccine strain (17D, a gift from professor Zhigang Yi of Fudan University) were propagated in Vero cells (ATCC CCL-81) at 37°C. Enterovirus 71 (EV71) was propagated in RD cells (ATCC CCL-136). Viral supernatants were clarified, concentrated, aliquoted, and stored at -80°C until use. Viral titers of HCVcc were determined by immunofluorescence, calculated and expressed as F.F.U./mL. WNV and YFV working stocks were titrated by plaque assay and are shown as plaque-forming units (PFU) per mL.

For virus infection assay, Huh7 or RD cells were seeded in 96-well plates and grown until 70-80% confluent. The cells were then infected with HCVcc, WNV, YFV and EV71 at a multiplicity of infection (MOI) of 1 for 1 h **in the presence or absence of** indicated concentrations of BEVs. After infection, free viruses were removed and the cells were then cultured in fresh complete medium for 24 hours. Subsequently, cells were subjected to immunofluorescence assay as previously described. Briefly, the cells were processed for immunofluorescence as previously described. Primary antibodies used here were a rabbit anti-HCV core polyclonal antibody (Abcam, ab2740), rabbit polyclonal antibody against the flavivirus NS1 protein or a rabbit anti-enterovirus 71 VP1 polyclonal antibody (GeneTex, GTX132339) for the corresponding virus. Infection rates were determined by fluorescence microscopy.

#### Multi‑generational colony morphology observation of *C. aquifrigidense* M24

#### The cryopreserved *C. aquifrigidense* M24 was revived by streaking onto a YEB solid medium plate and incubated at 28 °C for 24 h to obtain single colonies (recorded as passage 1). A single colony with typical morphology was picked from the passage 1 plate, serially diluted in 10‑fold steps to 10⁻⁶. Then, 20 μL of the diluted suspension was spread evenly onto the surface of a fresh YEB solid medium plate and incubated at 28 °C for 48 h to obtain passage 2. The same procedure was repeated for each subsequent passage to successively obtain passages 3, 4, 5, 6 and 7. After incubation, each plate was photographed at a similar height and angle using a digital camera (Canon, Japan). The images from all passages were saved and analyzed to compare overall colony morphology, size, color and surface characteristics.

#### 16S rDNA sequencing

#### The 16S rDNA of *C. aquifrigidense* M24 was amplified using high-fidelity PCR (Pfu from Promega, 30 cycles) with universal primers 27F and 1492R. The PCR product was then examined by 1% agarose gel electrophoresis and subsequently sent to Shanghai Sangon Biotech for sequencing. The resulting sequences were analyzed using BLASTn on the NCBI website, with the rRNA/ITS database selected. Homologous sequences were retrieved, and a molecular phylogenetic tree was reconstructed using MEGA7.0.

#### Gram staining of *C. aquifrigidense* M24

#### Gram staining of *C. aquifrigidense* M24 was performed using a Gram staining kit (Land Bridge, CM1001, Beijing) according to the manufacturer’s instructions. *Bacillus velezensis* (*B. velezensis*) and *Escherichia coli* (*E. coli*) were used as the positive and negative controls, respectively. A clean, grease‑free glass slide was prepared, and an appropriate amount of sterile water was placed at the center. A small amount of *C. aquifrigidense* M24 or control colonies was picked using an inoculation loop under aseptic conditions, transferred into the sterile water, and spread evenly to form a thin bacterial smear. After air‑drying and heat‑fixing over a flame, the bacteria on the slide were stained with crystal violet solution (0.5% w/v) for 1 minute at room temperature, then gently rinsed with a slow stream of distilled water until the run‑off was colorless. Iodine solution was added, and the smear was mordanted for 1 minute, followed by rinsing with distilled water as before. Then, 95% ethanol was added to decolorize for 0.5–1 minute, and the slide was immediately rinsed with distilled water to stop decolorization. Finally, basic fuchsin solution (0.5% w/v) was added to counterstain for 0.5 minute, followed by a rinse with distilled water. The bacteria were then observed, and images were captured under an oil immersion lens (100×) using a microscope (Nikon, Japan). Gram‑positive bacteria appear purple, while Gram‑negative bacteria appear red.

**Nano Flow Cytometry analysis**

The size and concentration of crude BEVs were characterized by Nano FCM. Briefly, the prepared BEVs samples were diluted (1:1000), stained with the dye DiO and then analyzed using the Flow Nano Analyzer (Apogee Flow Cytometer, China) according to the manufacturer’s instructions. The lasers were calibrated using 200-nm control beads, which were then regarded as a reference for particle concentration. In addition, a mixture of different-sized beads was analyzed to set a reference for size distribution. PBS was analyzed as a background signal. Particle concentration and size distribution were calculated using the nFCM software package, Nano FCM v. 2.0.

#### Observation of bacteria by Scanning Electron Microscopy (SEM)

To examine bacterial surface morphology, size, pili, flagella, and other structural features, bacteria cultured to the mid-log phase were collected, subjected to low-speed centrifugation, and washed with PBS. The samples were fixed overnight at 4°C with 2.5% glutaraldehyde in PBS. After rinsing, post-fixation was performed using 1% osmium tetroxide (in PBS) at 4°C in the dark for 1-2 hours. The fixed samples were then dehydrated through a graded ethanol series, critical-point dried, and coated with a 5-10 nm layer of gold or platinum using an ion sputter coater to enhance conductivity and secondary electron emission. Bacterial were observed under a scanning electron microscope at an accelerating voltage of 5-15 kV, and high-resolution images were acquired.

#### Viral binding assay to assess the effect of BEVs on DENV attachment

Viral binding assay was performed to evaluate the effect of BEVs on DENV attachment as previously described with some modifications. Huh7 cells were seeded in 24-well plates 24 hours prior to the experiment. The cell culture plate was then pre-chilled on ice, followed by the simultaneous addition of DENV (MOI=10) and the indicated concentrations of BEVs. The plate was incubated on ice for 1 hour to allow the virus particles to bind to cell surface while preventing viral internalization. After incubation, the supernatant was removed, and the cells were washed three times with ice-cold PBS. Total RNA was then directly extracted from the cells using TRIzol reagent (TaKaRa, 9109). The amount of cell-associated viral RNA, which corresponds to the bound virus, was quantified by quantitative real-time PCR (qRT-PCR).

#### Viral entry assay to analyze the effect of BEVs on DENV entry

Viral entry assay was performed following the initial binding step as previously described above. After virus binding on ice for 1 hour, Huh7 cells were rapidly transferred to 37°C for 1 hour to initiate viral internalization. After this incubation, the supernatant was removed, and the cells were washed three times with PBS to remove any non-internalized virus particles. The cells were then collected, and the inhibitory effect of BEVs on DENV entry was assessed by quantifying the internalized viral RNA using qRT-PCR. Intracellular RNA levels were expressed relative to the control.

#### Post-entry assay to assess the post-entry inhibitory effect of BEVs on DENV

Huh7 cells were infected with DENV (MOI=1) for 1 hour at 37°C. After the infection period, the inoculum was removed, and the cells were treated with different concentrations of BEVs for 1 hour at 37°C. Following BEV treatment, the medium was replaced with fresh complete medium, and the cells were cultured for an additional 24 hours. Then, the cells were harvested, and the inhibitory effect of BEVs on the post-entry stage of DENV infection was evaluated by quantifying viral RNA levels using qRT-PCR.

#### Cell viability Assay (CCK-8 method)

According to the manufacturer’s instructions, Huh7 cells were incubated with serially diluted supernatant from *C. aquifrigidense* M24 for 4 hours or BEVs for 2 hours. Subsequently, CCK-8 (2-(2-Methoxy-4-nitrophenyl)-3-(4-nitrophenyl)-5-(2,4-disulfophenyl)-2H-tetrazolium, monosodium salt) solution (Dojindo, CK04) was added (10 µL per well). After further incubation for 2 hours at 37°C, the absorbance at 450 nm was measured using a 96-well plate reader (BioTek, USA), and relative cell viability was calculated.

**Results**


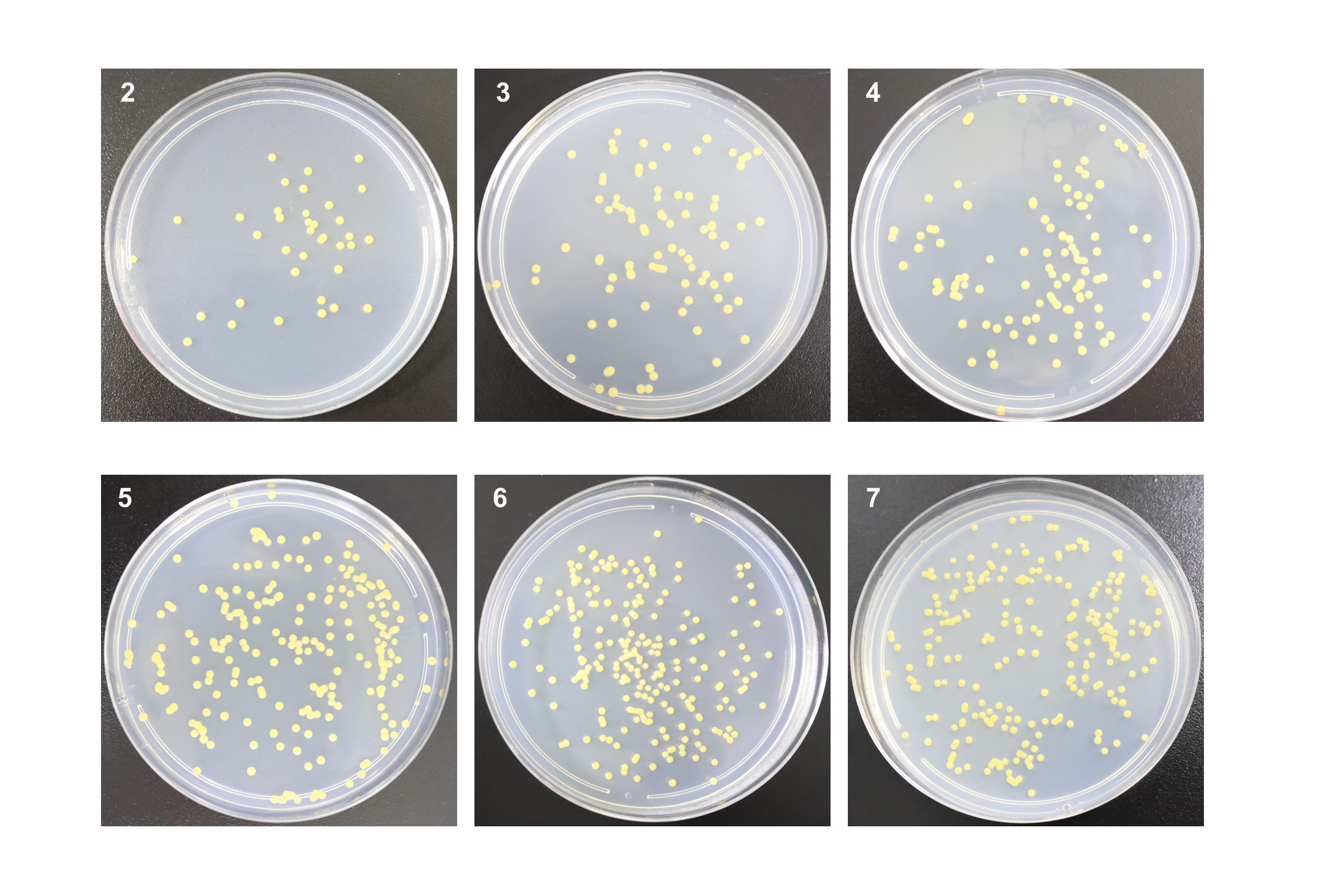


**Figure S1** Colony morphology of *C. aquifrigidense* M24 from passages 2 to 7 during serial subculturing. All colonies appear golden yellow, circular, moist, and smooth-surfaced. They are relatively uniform in size and evenly distributed as single, discrete colonies on the plates of passages 2–7. After seven consecutive passages, the colony color, size, and morphology remain stable.


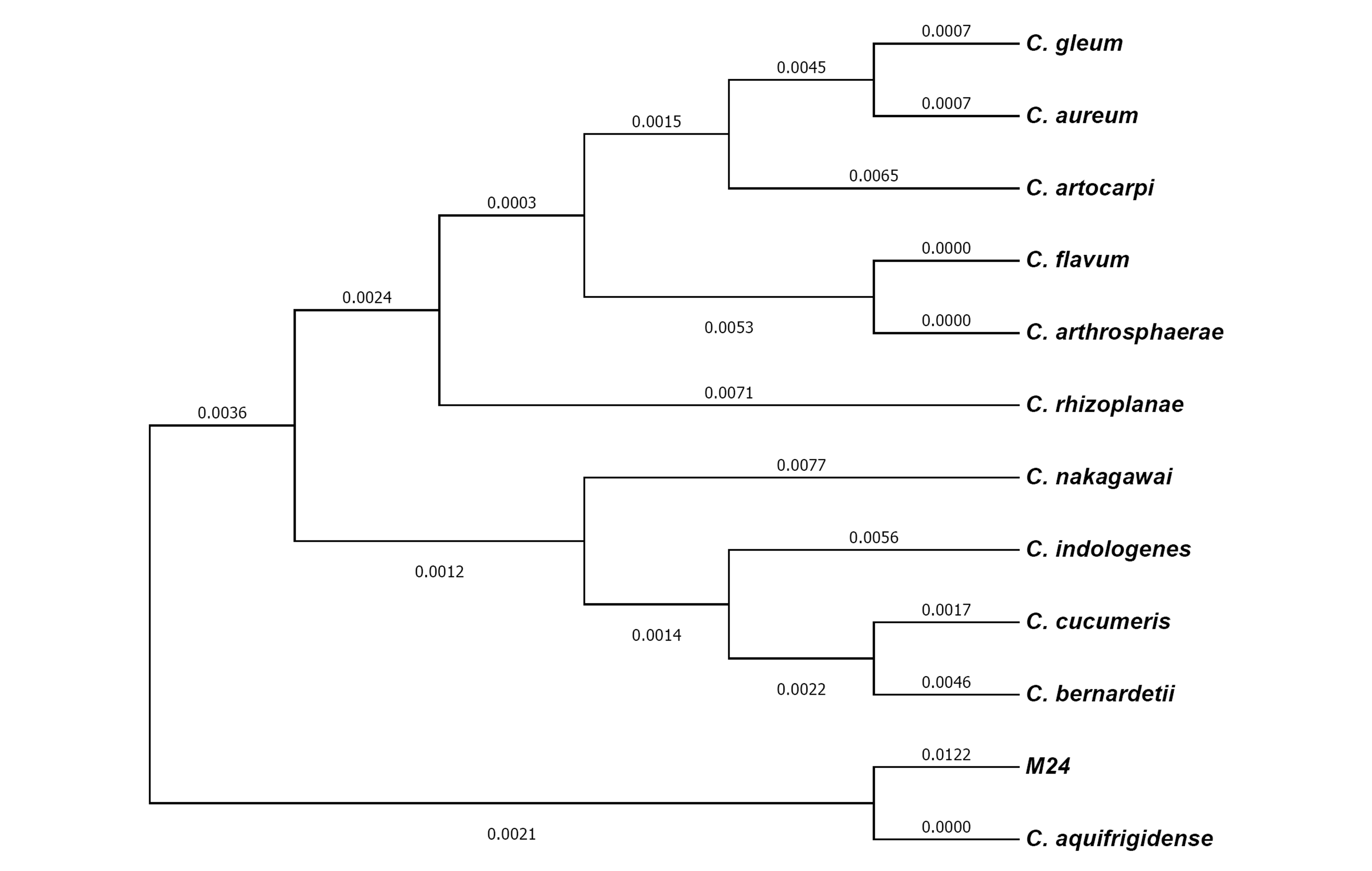


**Figure S2** Molecular phylogenetic tree of *Chryseobacterium*. The numbers in the figure represent evolutionary distances; the 16S rDNA sequences used were derived from *C. aquifrigidense* CW9 (accession no. NR_044334.1), *C. indologenes* NBRC 14944 (NR_044334.1), *C. flavum* CW-E 2 (NR_044334.1), *C. arthrosphaerae* CC-VM-7 (NR_044334.1), *C. gleum* NBRC 15054 (NR_113722.1), *C. cucumeris* GSE06 (NR_113722.1), *C. aureum* personal::is 17SE7 (NR_170500.1), *C. nakagawai* G41 (NR_170500.1), *C. artocarpi* UTM-3 (NR_170500.1), *C. bernardetii* G229 (NR_170500.1), and *C. rhizoplanae* JM534 (NR_170500.1).


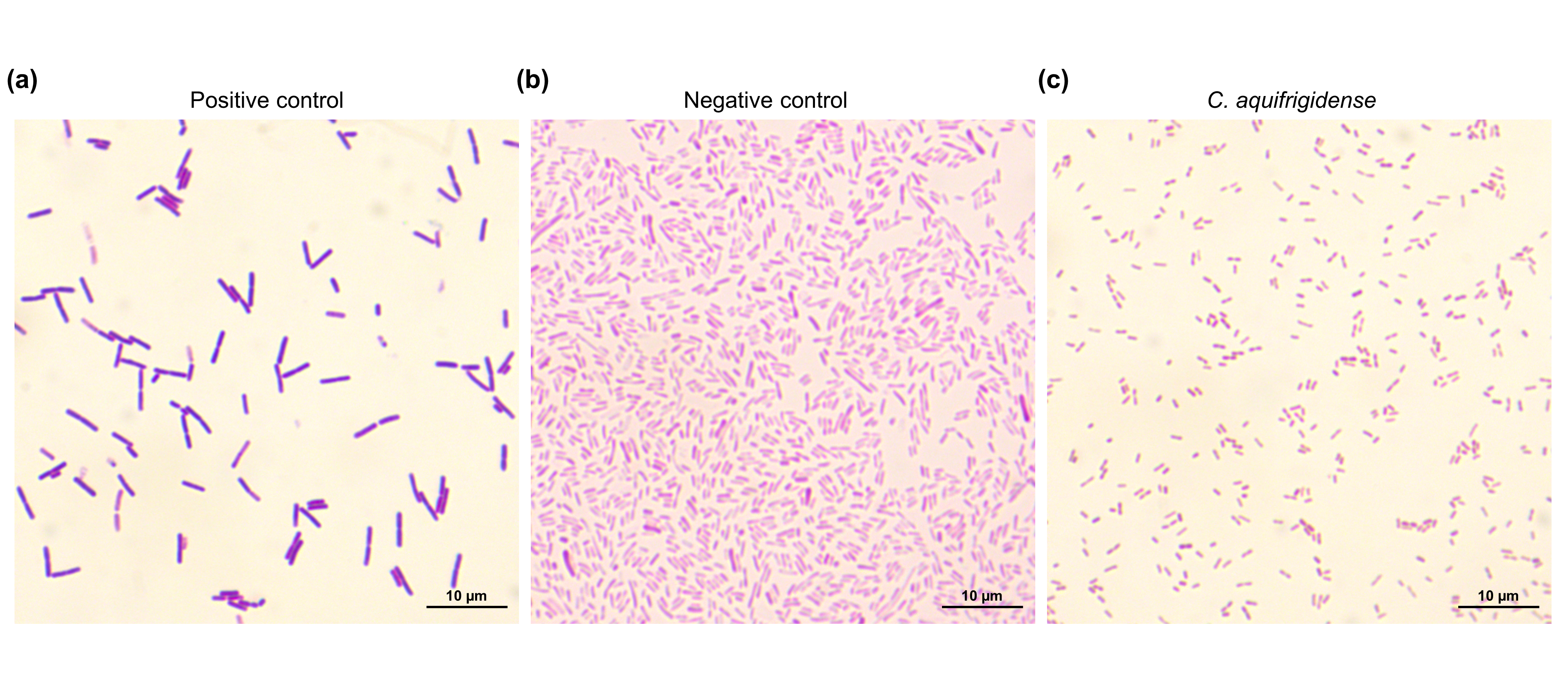


**Figure S3** Gram staining of *C. aquifrigidense* M24. (a) Cells of *B. velezensis* as the positive control appear purple (Gram‑positive). (b) Cells of *E. coli* as the negative control appear red (Gram‑negative). (c) Cells of M24 appear red, indicating that it is Gram‑negative. Scale bar = 10 μm.

**Figure S4** Representive immunofluorescence images showing the DENV infection by addition of different concentrations of filtered bacterial supernatant. Mock or the YEB medium treated cells were used as negative and positive control, respectively. Scale bar = 200 µm.


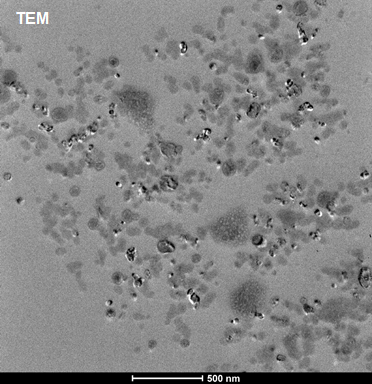


**Figure S5** Transmission electron microscopy (TEM) image showing the intercellular areas surrounding bacteria. Abundant membrane-derived vesicular structures and their aggregates are visible in these spaces. Scale bar = 500 nm.


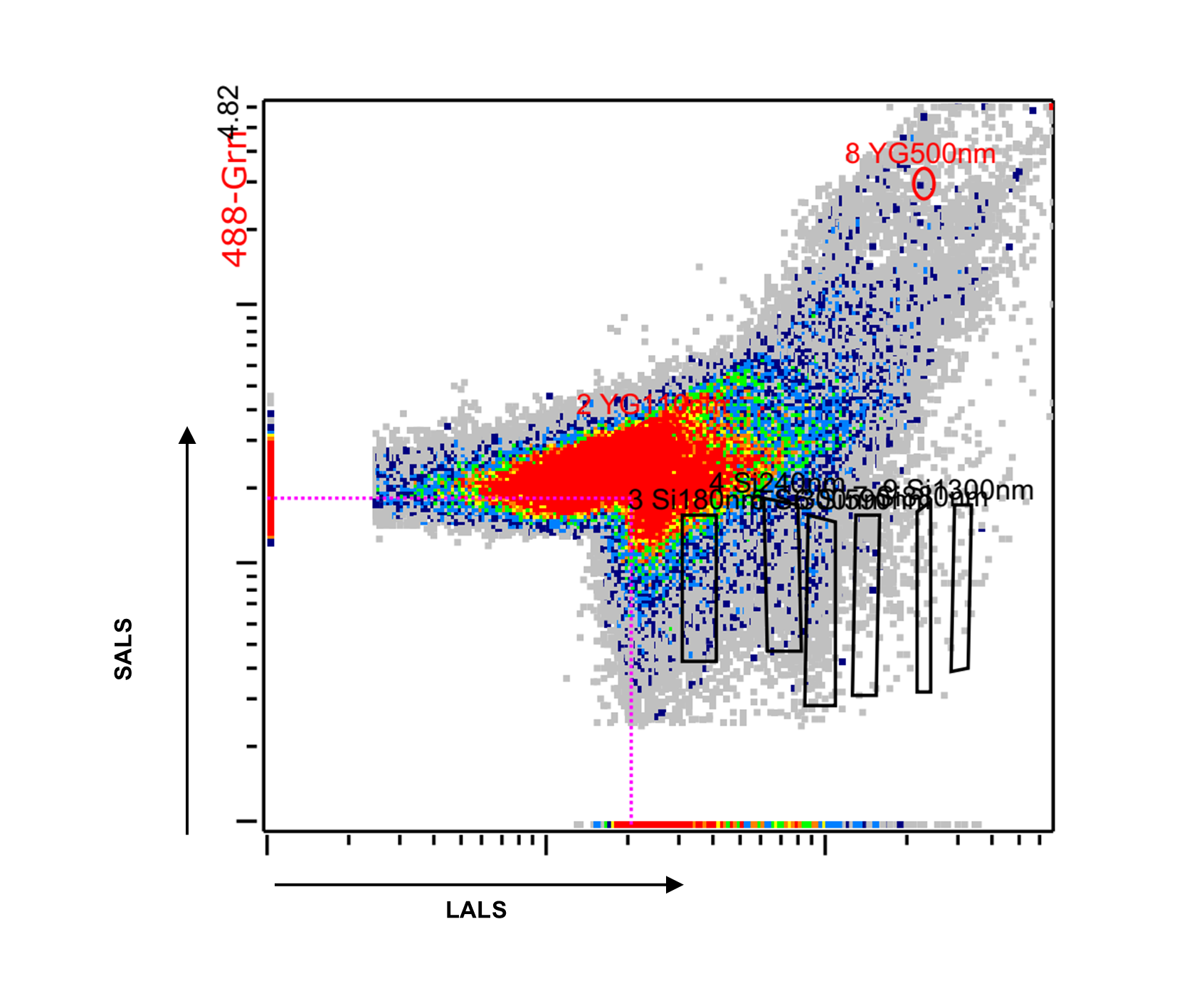


**Figure S6** Nanoparticle size distribution of the ultracentrifugation pellet staining with DiO, as detected by nano-flow cytometry.

**Figure S7** Effect of different concentrations of BEVs on the viability of cells, determined by CCK-8 assay. Data are presented as mean ± SD (n = 3 for each group); a one-way ANOVA test followed by Dunnett's multiple comparisons test, with the PBS group serving as the control group. ns, not significant.


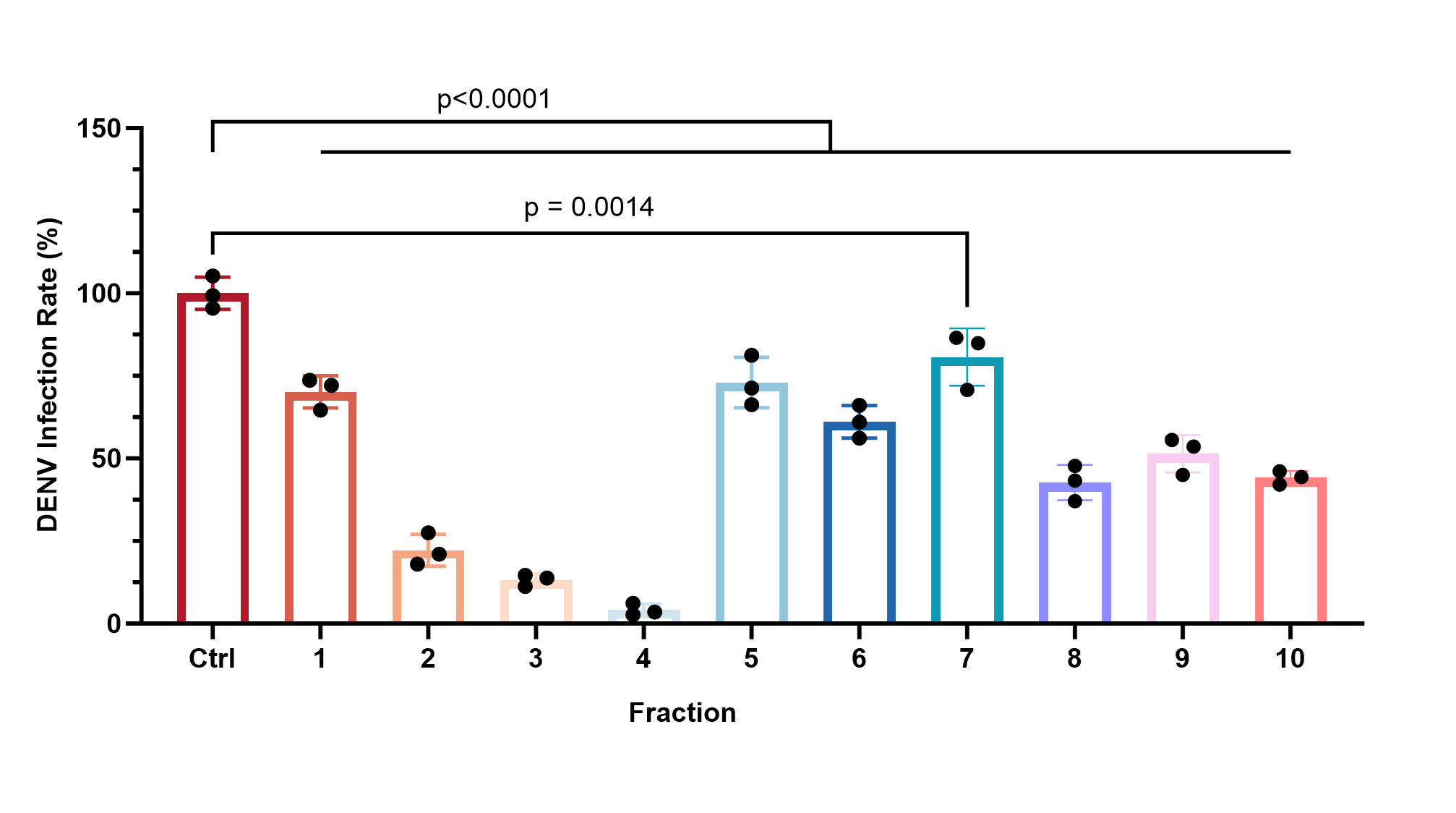


**Figure S8** Evaluation of anti-DENV activity of different fractions (F1-F10). The results show that fractions F3 and F4, at a final concentration of 25%, exhibited the most potent inhibition with rates exceeding 85%, indicating that the antiviral components were primarily enriched in these two fractions. Data are presented as mean ± SD (n = 3 for each group); one-way ANOVA test followed by Dunnett's multiple comparisons test, with the Ctrl group serving as the control group.
